# Interactions of Nedaplatin with Nucleobases and Purine Alkaloids: Their Role in Cancer Therapy

**DOI:** 10.3390/biomedicines13071551

**Published:** 2025-06-25

**Authors:** Kamil Szupryczyński, Beata Szefler

**Affiliations:** 1 Doctoral School of Medical and Health Sciences, Faculty of Pharmacy, Collegium Medicum, Nicolaus Copernicus University, Jagiellońska 13, 85-067 Bydgoszcz, Poland; 503592@doktorant.umk.pl; 2 Department of Physical Chemistry, Faculty of Pharmacy, Collegium Medicum, Nicolaus Copernicus University, Kurpińskiego 5, 85-096 Bydgoszcz, Poland

**Keywords:** Nedaplatin, purine alkaloids, cancer treatment, Gibbs free energy change (ΔGr), Density Functional Theory (DFT), UV-Vis spectroscopy

## Abstract

**Background**: Nedaplatin is a platinum-based anticancer drug that combines the benefits of Cisplatin and Carboplatin, retaining Cisplatin’s anticancer activity while reducing toxicity similar to Carboplatin. After hydrolysis, Nedaplatin targets purines in DNA and forms cross-links that induce cell death via apoptosis. However, it is important to consider how the presence of other chemical compounds with structural similarities to Adenine or Guanine, such as aromatic, purine, or pyrimidine compounds containing a nitrogen atom with a free electron pair, might influence its activity at the cellular level. Alkaloids with structures similar to DNA nucleobases are common, and their influence on Nedaplatin’s activity requires investigation. **Methods**: In this study, the interactions between Nedaplatin (including its hydrolyzed forms, such as [Pt(NH_3_)_2_(H_2_O)_2_]^2+^ and [Pt(NH_3_)_2_(H_2_O)(OH)]^+^) and nucleobases (Adenine and Guanine) and purine alkaloids (Caffeine, Theobromine and Theophylline) were thoroughly investigated using theoretical (density functional theory, DFT) and experimental (UV-Vis spectroscopy) methods. DFT calculations were performed at the B3LYP/6-31G(d,p)/LANL2DZ and MN15/def2-TZVP levels, with structure optimization and harmonic analysis in the gas phase and aqueous solution (modeled using IEF-PCM). UV-Vis spectroscopy was used to verify theoretical findings by examining changes in absorption spectra. **Results**: Both theoretical and experimental studies confirmed that Nedaplatin forms complexes with both nucleobases and purine alkaloids. Nedaplatin was found to exhibit a higher affinity for nucleobases than for purine alkaloids. Furthermore, this affinity was dependent on the computational method used and on the hydrolyzed form of Nedaplatin. Theoretical calculations showed the formation of stable complexes through bonding with nitrogen atoms in the ligand molecules, which was confirmed by changes in UV-Vis spectra, indicating adduct formation. **Conclusions**: The results indicate that Nedaplatin readily forms complexes with both nucleobases and purine alkaloids, showing a stronger affinity for nucleobases. This finding highlights the potential importance of Nedaplatin’s interactions with other compounds present in the body, which may influence its effectiveness and mechanism of action in cancer therapy. These studies provide new insights into the molecular mechanisms of Nedaplatin’s action and may contribute to a better understanding of its pharmacological interactions. However, research requires confirmation not only in in vivo studies but also in clinical trials.

## 1. Introduction

**Figure 1:**
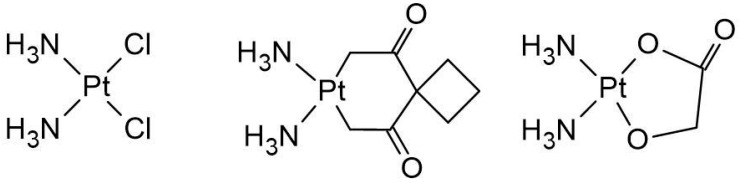
Structure of Cisplatin, Carboplatin, and Nedaplatin [1].

Nedaplatin is a second-generation platinum-based anticancer drug developed by Shionogi Pharmaceutical Company, first introduced in 1983 and approved for clinical use in Japan in 1995 (Figure 1) [1]. It is designed to combine the beneficial therapeutic effects of Cisplatin and Carboplatin while reducing the associated toxicities. Specifically, Nedaplatin demonstrates comparable anticancer efficacy to Cisplatin while exhibiting lower nephrotoxicity and gastrointestinal toxicity, marking it as a vital option in cancer therapy [2]. Myelosuppression, particularly thrombocytopenia, neutropenia, and anemia, remains a significant side effect and the most common dose-limiting factor [3].

**Figure 2:**
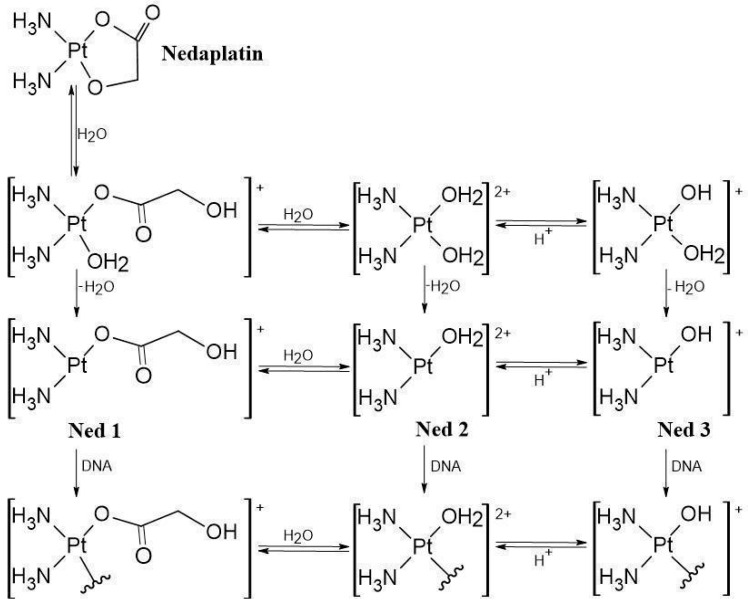
Hydrolysis of Nedaplatin: N1 to N3 represent the hydrolysis products [1].

Nedaplatin is administered intravenously as a prodrug that is inactive in the bloodstream due to the high concentration of chloride ions [4]. However, once Nedaplatin enters the cell through various transporters, including copper transporters [5], it is activated by hydrolysis, resulting in the formation of three active hydrolysis products [(NH_3_)_2_Pt(H_2_O)(OCOCOH)]^+^, [(NH_3_)_2_Pt(H_2_O)_2_]^2+^ and [(NH_3_)_2_Pt(H_2_O)(OH)]^+^ referred to in the article as products N1, N2, and N3 [1,6] (Figure 2). These active metabolites preferentially target the nitrogen atoms in DNA, with the N7 position of Guanine and Adenine being the most favored sites [7,8,9,10]. Additionally, they can interact with proteins [11,12,13,14]. These interactions occur because the platinum atom in Nedaplatin is highly electrophilic, which makes it attracted to nucleophiles, such as nitrogen or sulfur atoms that possess a lone pair of electrons [7,8,9,10,11,12,13,14,15,16].

**Figure 3:**
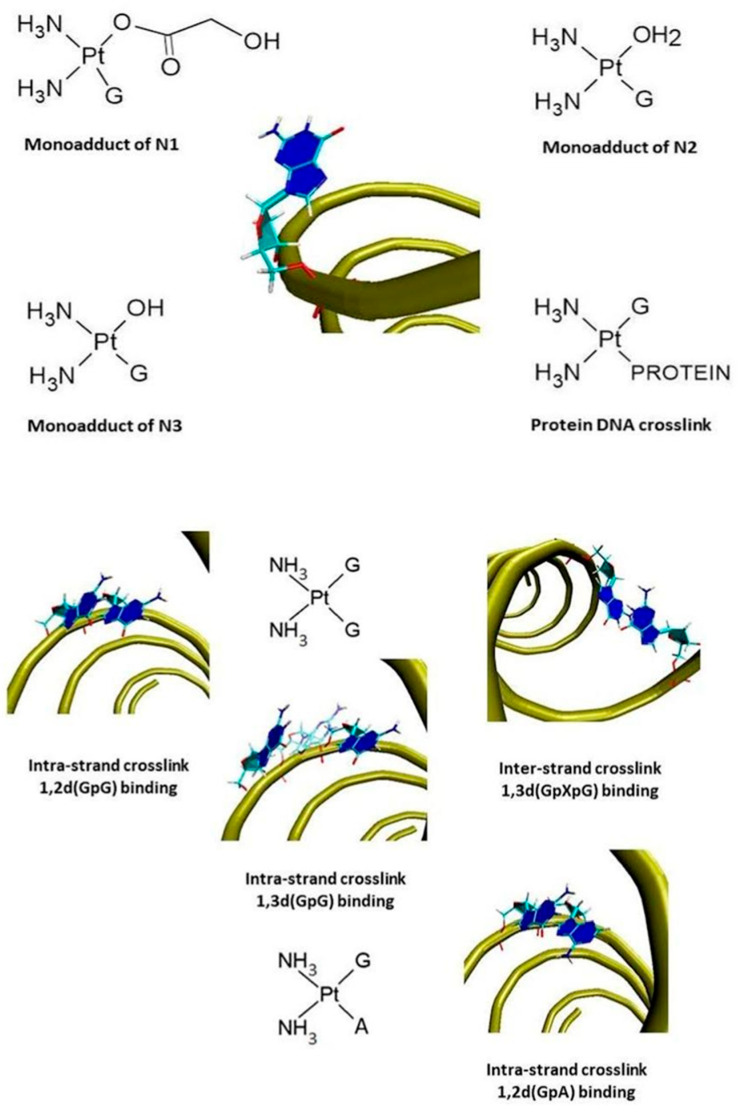
Monoadducts of N1–N3 and diadducts of N2 as products of hydrolysis of Nedaplatin with DNA [7].

This interaction leads to DNA distortions [17,18] by binding to monoadduct strands [19]. For the N2 product of hydrolysis of Nedaplatin, it can result in the formation of multiple DNA cross-links. A significant majority of these connections are intra-strand, primarily occurring between adjacent Guanine residues (1,2-d(GdG) binding) or with a one-base gap between them (1,3-d(GpG) binding) (Figure 3). Less frequently, but still often, cross-links can form between adjacent Guanine and Adenine residues (1,2-d(GpA) binding), as well as less common inter-strand and protein-DNA cross-links [20,21,22,23,24,25,26]. Moreover, Nedaplatin induces oxidative stress in cells by stimulating the production of reactive oxygen species [27,28,29,30,31,32]. If these changes are not repaired by the cell, they can lead to apoptosis [6,33,34,35,36].

Unlike Carboplatin, the dosage of Nedaplatin is based on body surface area rather than AUC (Area Under the Curve) [37,38,39]. AUC is a common pharmacokinetic term used to describe the total exposure of the body to a drug over time. It is calculated from the concentration-time curve after the drug is administered and provides a measure of how long and how much of the drug is present in the bloodstream [37,38,39]. The recommended therapeutic dose is 80–100 mg/m^2^ every 4 weeks, along with a repeated infusion of 1000 mL [2]. The pharmacokinetic profile of Nedaplatin is comparable to that of Carboplatin, but its affinity for binding to plasma proteins is lower than that of Cisplatin [40]. Consequently, the drug has a shorter half-life in the bloodstream (1.1–4.4 h), which allows for its rapid elimination from the body [1].

Nedaplatin has been used in the treatment of various cancers, including non-small-cell lung cancer [41,42,43,44,45,46,47], neuroendocrine lung carcinoma [48], squamous cell lung cancer [49,50,51,52], lymphoepithelioma [53], urothelial cancer [54,55,56], esophageal esophageal [57,58], head and neck cancer [59], esophageal squamous cell carcinoma [60,61,62,63,64], and ovarian cancer [65].

**Figure 4:**
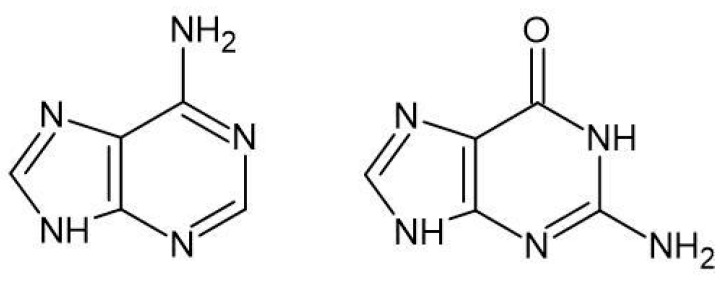
Structures of nucleobases: Adenine (A) and Guanine (G).

Nedaplatin is administered as a prodrug in an intravenous solution. Once inside the cell, it is hydrolyzed into its active forms. These active forms interact with nucleophiles in the cytoplasm, such as cysteine-containing proteins, including glutathione, generating reactive oxygen species and increasing oxidative stress [27,28,29,30,31,32]. Most importantly, the active forms of Nedaplatin bind to nucleic acids, primarily at the N7 positions of Guanine and Adenine in DNA (Figure 4) [1,7]. As a consequence of this binding to nucleobases, cross-links are formed both between DNA strands and within a single strand. If the changes induced by Nedaplatin are not repaired, they can lead to spontaneous cell death through apoptosis [6,33,34,35,36].

The previous study [66,67,68,69,70] demonstrated that platinum compounds can react with structures similar to nucleobases that contain a nitrogen atom with a lone pair of electrons in the aromatic rings. Theoretical and experimental research confirmed that structures such as B vitamins can readily react with chemotherapeutics containing platinum atoms in their structures [66,67,68,69,70].

**Figure 5:**
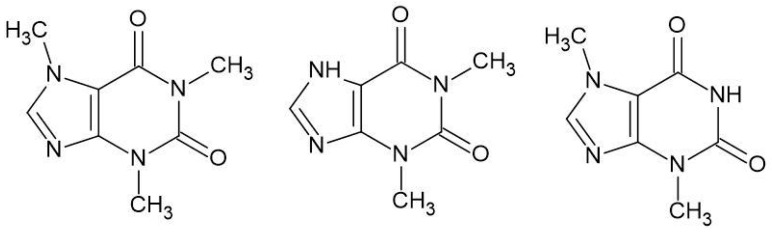
Structures of alkaloids: Caffeine (Caf), Theophylline (Tep), and Theobromine (Teb).

A related group of drugs is purine alkaloids (Figure 5), specifically methylxanthines, which include three naturally active substances: Caffeine, Theophylline, and Theobromine. The first two are found in beverages such as coffee and tea, while Caffeine and Theobromine are present in cocoa. Methylxanthines have a multifaceted effect on the body; for instance, they competitively inhibit phosphodiesterase types III and IV (PDE), an enzyme responsible for the hydrolysis of cyclic AMP in smooth muscle cells, leading to bronchodilation [71]. They stimulate the central nervous system (CNS) and can cause muscle tremors, excitability, sleep disturbances, and increased respiration. Additionally, they exert a positive inotropic and chronotropic effect and cause vasodilation [72]. Furthermore, they have a mild diuretic effect [73].

Theophylline and its derivative, Aminophylline, are commonly used in treatment. Theophylline is effective for both acute asthma attacks and chronic asthma management, as well as for reducing the severity of COPD symptoms [74,75]. Both Theophylline and Aminophylline can be administered orally or intravenously. Theophylline works by relaxing the smooth muscles of the bronchi and pulmonary blood vessels, and it decreases the respiratory tract’s reactivity to histamine, methacholine, and allergens [74,75]. Theophylline binds to the adenosine A2B receptor, blocking adenosine-mediated bronchoconstriction [74,75].

Caffeine is used to treat primary apnea in premature infants, typically in the form of citrate [76]. Furthermore, Caffeine can be combined with other medications, such as dimenhydrinate (which is used to prevent and treat motion sickness), to reduce drowsiness and with acetaminophen (which has analgesic, antipyretic, anti-inflammatory, and platelet aggregation-inhibiting properties) to enhance its effectiveness [77,78,79].

On the other hand, Theobromine has been utilized as both a bronchodilator and a vasodilator. It has weaker diuretic and smooth muscle-relaxing effects than Theophylline and does not affect the central nervous system. Historically, Theobromine was used as a diuretic and in the treatment of angina and hypertension [80,81].

Alkaloids represent an intriguing group of compounds that should be considered in two contexts: as active ingredients in pharmaceuticals and as constituents of food. Unfortunately, the impact of purine alkaloids is often overlooked. While there is considerable awareness regarding drug interactions, few people consider how a cup of coffee or a bar of chocolate might influence cancer treatment. It is important to note that coffee is the world’s most popular beverage after water [77], and its primary component, Caffeine, may potentially interact with Nedaplatin.

The aim of this study is to determine the binding affinity of Nedaplatin to purine alkaloids, with particular emphasis on Caffeine, Theobromine and Theophylline, using UV-Vis spectroscopy and quantum chemical calculations. We hypothesize that Nedaplatin may exhibit significant binding affinity to alkaloids, which could potentially influence its anticancer activity.

The binding interactions will be analyzed through theoretical calculations using the B3LYP/6-31G(d,p) and MN15/def2-TZVP methods, focusing on the Gibbs free energy changes (ΔGr) associated with the formation of complexes. We will also compare these theoretical results with experimental UV-Vis spectroscopy findings to ascertain the stability and formation of the complexes over time. However, the results of the experimental UV-Vis studies will be compared with the theoretical ones.

By addressing these objectives, this study provides preliminary insights into the binding mechanisms of Nedaplatin and its potential molecular interactions with purine alkaloids commonly found in the human diet. Although the pharmacological interactions of anticancer drugs are well characterized, the influence of dietary compounds, such as Caffeine, Theobromine, and Theophylline, on the behavior of chemotherapeutics like Nedaplatin remains insufficiently explored.

This investigation is relevant in the context of growing interest in drug–diet interactions; however, it must be emphasized that the current results are limited to computational and in vitro spectroscopic data. No clinical or pharmacokinetic evidence is currently available to confirm that these dietary alkaloids interfere with Nedaplatin’s therapeutic activity. Therefore, the findings of this study should be viewed as hypothesis-generating rather than conclusive.

In conclusion, this study explored the potential binding affinity between Nedaplatin and selected purine alkaloids. While the results may help inform future research on Nedaplatin’s pharmacodynamics and interactions with dietary components, further in vivo studies and clinical validation are required to assess the biological and therapeutic significance of these interactions.

## 2. Materials and Methods

### 2.1. Theoretical Study

The experimental methodology was based on Density Functional Theory (DFT) [82,83] to model and analyze the geometric, energetic, and electronic properties of the investigated compounds. The theoretical models were constructed using GaussView 6.0.16 software [84]. which facilitated the visualization and initial setup of molecular structures. Quantum-chemical computations were carried out with the Gaussian 16 Rev. C.01 software package [84,85].

To locate energy minima and optimize molecular geometries, two levels of theory were employed:

- B3LYP/6-31G(d,p) [86,87]: This hybrid functional combines Becke’s 1988 exchange functional [88] with the Lee–Yang–Parr correlation functional [89], incorporating some Hartree–Fock exchange. It is widely used for its versatility across various molecular systems. For heavy atoms such as platinum, the LanL2DZ basis set was used [90,91], which includes relativistic effective core potentials to account for heavy-element effects.
- MN15/def2-TZVP [92,93]: This functional is designed for complex systems with strong electronic interactions and larger molecular sizes. It employs a different exchange-correlation approach and provides higher accuracy and universality, especially for systems with multiple electrons.

Energy minimization at both levels was performed to identify stable structures. To accurately calculate Zero Point Energies (ZPE), harmonic vibrational frequency calculations were conducted. These calculations also helped verify that the optimized structures correspond to true minima on the potential energy surface (no imaginary frequencies were found). It should be noted that only one tautomeric and conformational form of each purine alkaloid was considered in this study. While this approach ensures consistency in comparing binding affinities, the potential influence of other tautomers or conformers is acknowledged as a limitation and will be addressed in future work.

The solvent effects of water were modeled using the Polarizable Continuum Model (IEF-PCM), with radii based on the Bondi scheme [94], to simulate an aqueous environment and account for solvation effects. This implicit solvation model allows for the efficient approximation of bulk aqueous effects by treating the solvent as a polarizable dielectric continuum, which is appropriate for assessing relative affinities in a computationally tractable manner. While this model does not capture specific solute–solvent interactions such as hydrogen bonding, it provides a reasonable compromise between accuracy and computational cost for the comparative analysis performed in this study [95,96,97,98,99].

Spectroscopic properties were computed using the PBE0 functional, a hybrid approach known for reliable predictions of electronic spectra [40,100].

This methodology, combining different functionals and basis sets, was chosen to ensure accurate and comparable results, aligning with previous studies such as those on Cisplatin and nucleobases, where the B3LYP method provided results consistent with experimental data [91]. The use of multiple computational levels allows for comprehensive analysis and validation of the theoretical models [67,69,70].

### 2.2. Experimental Study (UV-Vis Spectroscopy)

The methodology for the experiment involved analyzing the interactions between B-group vitamins, nucleobases, and Oxaliplatin using UV-Vis spectroscopy. The spectroscopic measurements were conducted with a Biosens UV-6000 spectrophotometer (Warsaw, Poland) over a wavelength range of 190 nm to 500 nm.

Reagents used in the study were sourced from different suppliers: Caffeine (purity 99.7%) was obtained from Thermo Scientific (Nanjing, Jiangsu), China; Theophylline (1,3-Dimethylxanthine, 98%) and Theobromine (3,7-Dimethyl-3,7-dihydro-1H-purine-2,6-dione, 99%) from Angene; Nedaplatin (purity 98%) from AmBeed (Buffalo Grove, IL, USA).

All UV-Vis experiments in this study were performed in phosphate buffer at pH 7.4 to reflect physiological plasma conditions. However, it is important to note that the tumor microenvironment is typically more acidic, with pH values ranging between 6.5 and 6.8. Previous studies have demonstrated that platinum-based compounds can undergo pH-dependent changes in hydrolysis and reactivity [31].

For sample preparation, a stock solution of Nedaplatin was prepared in phosphate buffer (pH 7.4) at a concentration of 3.23 × 10^−6^ mol/L. Mixtures of nucleobases or vitamins with Nedaplatin were then prepared by adding an alkaloid in a molar ratio of 1:2 (Nedaplatin to alkaloid), resulting in a final alkaloid concentration of 6.46 × 10^−6^ mol/L.

The concentration of Nedaplatin used in this study (3.23 × 10^−6^ mol/L) was s elected to reflect clinically relevant plasma levels. Pharmacokinetic studies report that following standard therapeutic dosing (80–100 mg/m^2^), Nedaplatin reaches peak plasma concentrations ranging from 2 to 10 μg/mL (approximately 5.3 × 10^−6^–2.6 × 10^−5^ mol/L), depending on patient-specific parameters such as renal clearance [21,40]. Therefore, the concentration used in our in vitro experiments is within the physiologically relevant range, particularly when considering dilution factors in extracellular environments.

The mixtures of Nedaplatin and alkaloid (nucleobase) were incubated at 37 °C. Samples were collected at specific time intervals: 0, 3, 12, 24, 36, and 168 h. Control samples containing only Nedaplatin, Caffeine, Theophylline, or Theobromine at identical concentrations dissolved in phosphate buffer (pH 7.4) were also prepared for comparison.

During measurements, the maximum absorption wavelengths for each mixture were identified to monitor potential interactions over time. This approach allowed for the assessment of how B-group vitamins and nucleobases influence the spectral properties of Nedaplatin under physiological conditions.

## 3. Results and Discussion

### 3.1. In Silico Study

Based on theoretical studies, the affinity of hydrolysis products of Nedaplatin (N1–N3) was analyzed concerning compounds belonging to the group of purine alkaloids, such as Caffeine (Caf), Theobromine (Teb), and Theophylline (Tep). This was compared to the affinity of the nucleobases Adenine (A) and Guanine (G).

**Figure 6:**
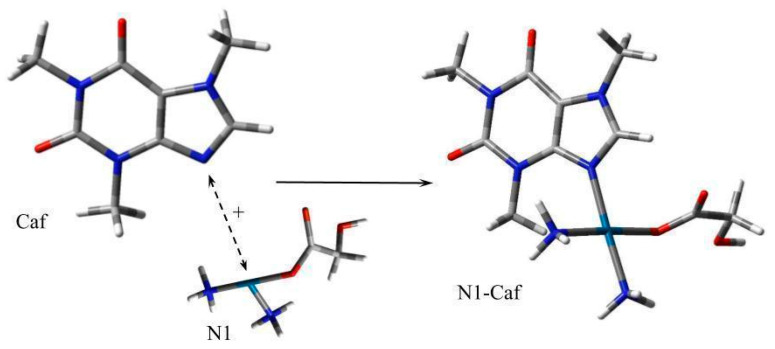
Scheme for formation of an alkaloids (Caf) complex with Nedaplatin (N1). The nitrogen atom (N) is marked in blue, the oxygen atom (O) in red, and the carbon atom (C) in gray, respectively.

For this analysis, the values of the Gibbs Free Energy changes (ΔG_rs_) were calculated for each complex. The mechanism of complex formation is consistent and involves the formation of a bond between the nitrogen atom of the nucleobase or alkaloid and the platinum atom of the active form of Nedaplatin (Figure 6).

**Figure 7:**
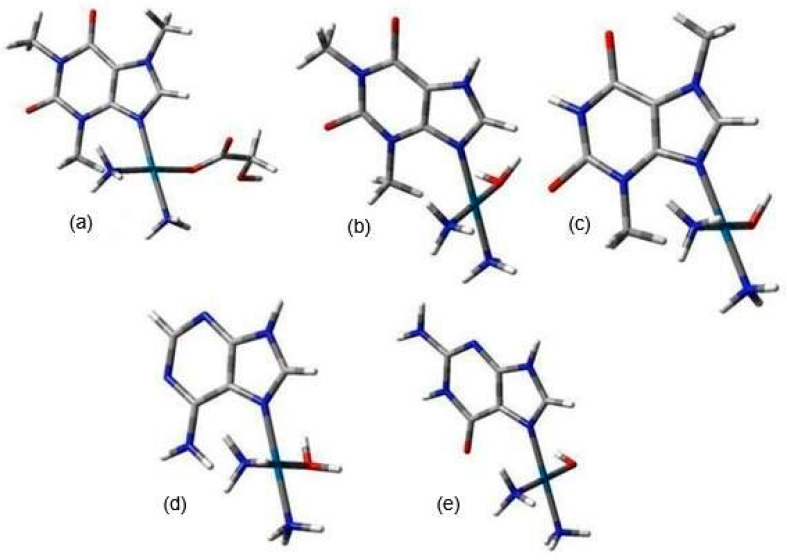
The examples of graphical representation of Nedaplatin (N1, N2, N3) complexes, calculated at the B3LYP/6-31G(d,p) and at MN15/def2-TZVP levels of theory, (**a**) N1-Caf, (**b**) N2-Tep, (**c**) N3-Teb, (**d**) N2-A, (**e**) N3-G. All structures are presented in Figure S1 in the Supplementary Materials. The nitrogen atom (N) is marked in blue, the oxygen atom (O) in red, and the carbon atom (C) in gray, respectively.

Since both alkaloids and nucleobases contain a lone pair of electrons in their aromatic rings, they can compete for binding to the active forms of Nedaplatin. Figure 7 illustrates the structures of the nedaplatin–alkaloid and nedaplatin–nucleobase complexes.

Table 1 presents the values of the change in Gibbs Free Energy (ΔG_rs_) for the reactions of the complexes formed between the subsequent hydrolysis products of Nedaplatin and nucleobases, as well as alkaloids. These values were calculated at two levels of calculations, B3LYP/6-31G(d,p)/LANL2DZ and MN15/def2-TZVP.

As shown in Table 1, the estimated ΔG_rs_ values are negative for both nucleobases and all purine alkaloids. However, the ΔG_rs_ values for the nedaplatin–alkaloid complexes are higher compared to those for the nedaplatin-nucleobase complexes. For instance, N1 in Table 1 indicates that the ΔG_rs_ values for reactions involving nucleobases are −22.9 and −20.6, whereas, for the alkaloids, they are −14.7, −15.7, and −15.3, respectively. These results suggest that N1 exhibits stronger affinities for nucleobases than for alkaloids.

**Figure 8:**
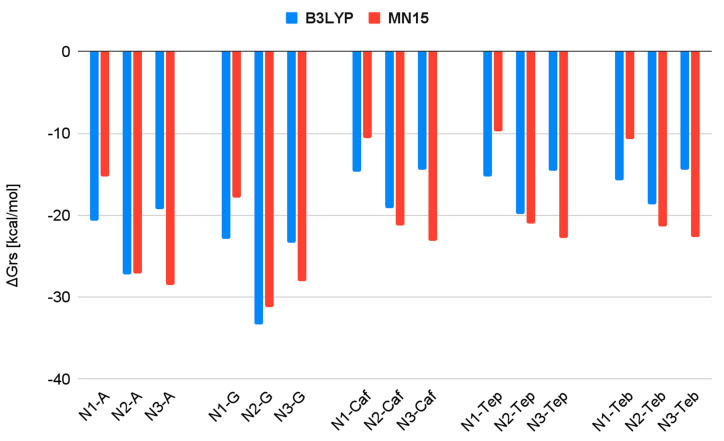
The ΔG_rs_ values for complexation. The blue and red colors represent the results obtained using the B3LYP and MN15 methods, respectively.

**Figure 9:**
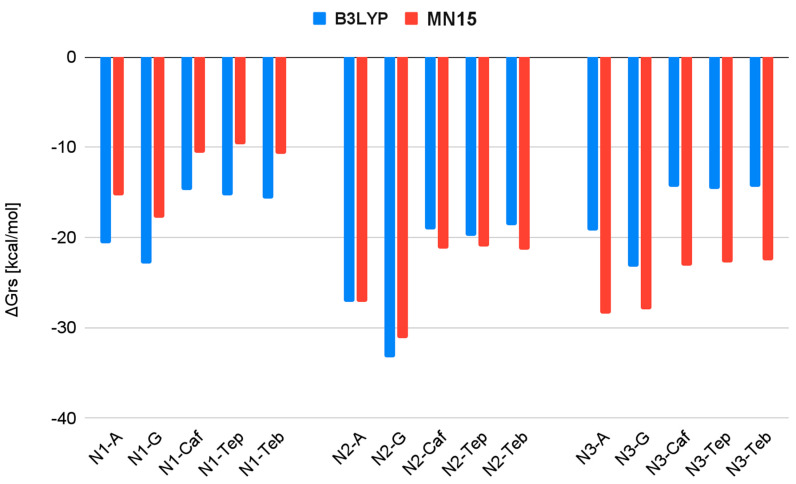
The ΔG_rs_ values for complexation, categorized by the active forms of Nedaplatin. The blue and red colors indicate the results obtained using the B3LYP and MN15 methods, respectively.

As shown in Figure 8 and Figure 9, all calculated ΔG_rs_ values are negative at both the B3LYP and MN15 levels of calculation, indicating the favorable formation of Nedaplatin hydrolysis products in association with nucleobases and purine alkaloid complexes. These results may support the therapeutic properties of Nedaplatin in its interactions with nucleobases; however, confirmation through clinical trials is necessary.

Among all tested substances, Nedaplatin exhibits the highest affinity for nucleobases (Figure 8), particularly for Guanine, for which it attains the lowest ΔG_rs_ values. However, for the complexes N3-guanine and N3-adenine optimized at the MN15/def2TZV level of calculation, the values of the delta Gibbs Free Energies are quite similar.

Among the hydrolysis products of Nedaplatin, N2 demonstrates the strongest affinity at the B3LYP/6-31G(d,p)/LANL2DZ level, while at the MN15/def2TZV level of calculation, both N2 and N3 hydrolysis products exhibit strong affinity (Figure 9).

Increasing the level of calculation alters the values of delta Gibbs Free Energy. The most significant increase was observed for product N3 when the calculation level was raised.

Conversely, a decrease was noted for N1 concerning all tested substances (Figure 9).

**Figure 10:**
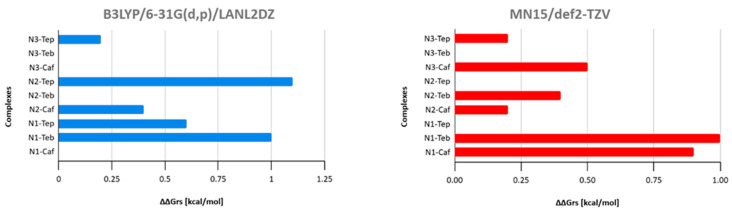
ΔΔGrs values of nedaplatin–alkaloid complexes are divided into the active forms of Nedaplatin: N1, N2, and N3. The blue and red colors represent the results obtained using the B3LYP and MN15 methods, respectively.

Figure 10 presents the ΔΔG_rs_ values for the nedaplatin–alkaloid complexes, based on the active form of Nedaplatin, across two different computational approaches. The ΔΔG_rs_ values were determined by subtracting the ΔG_rs_ value of the most stable (lowest-energy) nedaplatin–alkaloid complex from the ΔG_rs_ values of each complex within the same group. This comparison highlights the relatively small differences in binding energy between the alkaloids, as indicated by the ΔΔG_rs_ values, which range only from 0 to 1.1 kcal/mol. Such a narrow range suggests that the stability of Nedaplatin interactions with each alkaloid is quite uniform, reflecting a similar binding affinity across all alkaloids.

### 3.2. Experimental Analysis

To validate the in silico studies, an experimental study was conducted using UV-Vis spectroscopy. The quantum-mechanical calculations indicate the formation of nedaplatin-alkaloid complexes with Caffeine, Theophylline, and Theobromine, suggesting that all alkaloids exhibit a similar affinity for Nedaplatin. All UV-Vis experiments in this study were performed in phosphate buffer at pH 7.4 to reflect physiological plasma conditions. However, it is important to note that the tumor microenvironment is typically more acidic, with pH values ranging between 6.5 and 6.8. Previous studies have demonstrated that platinum-based compounds can undergo pH-dependent changes in hydrolysis and reactivity [31].

**Figure 11:**
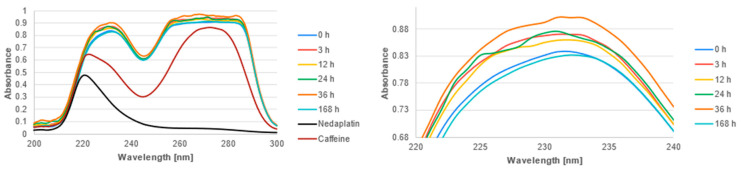
UV-vis absorbance spectrum of a mixture of Caffeine and Nedaplatin during incubation at 37 °C in phosphate buffer at pH 7.4.

**Figure 12:**
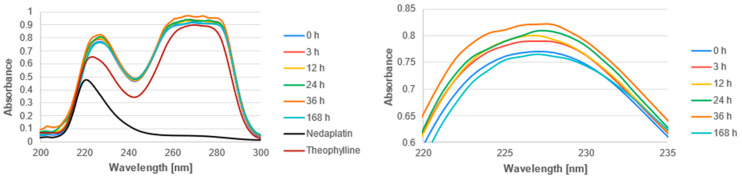
UV-vis absorbance spectrum of a mixture of Theophylline and Nedaplatin during incubation at 37 °C in phosphate buffer at pH 7.4.

**Figure 13:**
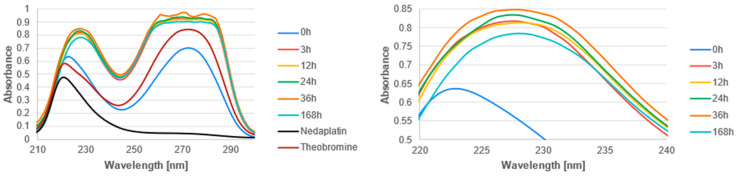
UV-vis absorbance spectrum of a mixture of Theobromine and Nedaplatin during incubation at 37 °C in phosphate buffer at pH 7.4.

**Figure 14:**
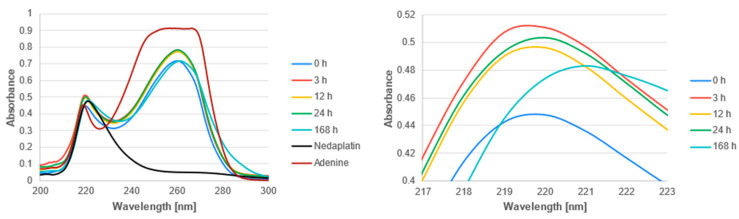
UV-vis absorbance spectrum of a mixture of Adenine and Nedaplatin during incubation at 37 °C in phosphate buffer at pH 7.4.

**Figure 15:**
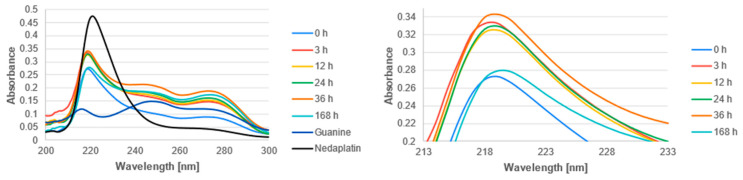
UV-vis absorbance spectrum of a mixture of Guanine and Nedaplatin during incubation at 37 °C in phosphate buffer at pH 7.4.

The UV-Vis spectra of the studied complexes were recorded over a wavelength range of 200 nm to 300 nm (Figure 11, Figure 12 and Figure 13). The obtained experimental UV-Vis spectra for the nedaplatin-alkaloid complexes were compared with the spectra for the nedaplatin-nucleobase (Adenine, Guanine) complexes (Figure 14 and Figure 15). Additionally, absorption spectra were obtained for pure Nedaplatin (shown in black), as well as for the individual alkaloids and nucleobases (shown in brown) in phosphate buffer. Absorption maxima were determined for all mixtures.

As illustrated in Figure 11, Figure 12 and Figure 13, the absorbance values for both the nedaplatin–alkaloid complexes and the individual alkaloids displayed two absorbance maxima at approximately 225 nm and 270 nm. In contrast, Nedaplatin itself exhibited only one maximum at around 220 nm. The overlap of the maxima from the alkaloids and the complexes hindered the ability to track the reduction in alkaloid concentration, thereby making it impossible to calculate the percentage of alkaloids that reacted with Nedaplatin.

**Figure 16:**
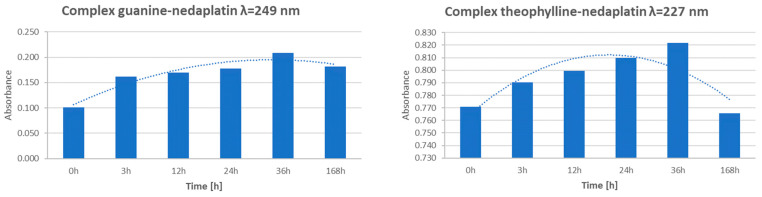
Examples of changes in maximum absorbance over time for Nedaplatin complexes with nucleobase (Guanine) or alkaloid (Theophylline) at a specifically defined wavelength (λ [nm]) are presented. The remaining research results can be found in Figures S2 and S3 in the Supplementary Materials.

The graphs reveal a consistent increase in absorbance during the first 36 h of incubation, indicating the progressive formation of complexes. However, after 168 h, a significant decrease in absorbance was observed, suggesting degradation or dissociation of the complexes (Figure 16). This observation indicates that the formed complexes are inherently unstable over extended periods.

Furthermore, the absorbance profiles for Caffeine, Theophylline, and Theobromine demonstrated remarkable similarity over time in both their uncomplexed and complexed states, highlighting that these alkaloids possess comparable physicochemical properties.

The obtained experimental UV-VIS spectra for nedaplatin-alkaloid complexes were compared with the experimental spectra for nedaplatin-nucleobase (Adenine, Guanine) complexes. Both Adenine and Nedaplatin showed a maximum absorbance at around 220 nm. However, Adenine also exhibits a second maximum at about 260 nm. Unfortunately, it was not possible to determine a clearly defined maximum, as the decrease in the concentration of Adenine causes the disappearance of the peak at a wavelength of 220. The conclusions regarding the complexation process and the decay over time for the nedaplatin-adenine complex are similar to those for the nedaplatin–alkaloid complexes. Specifically, a rise in the maximum absorbance over time was first observed, suggesting the formation of the nedaplatin–adenine complex, followed by a decrease in the maximum absorbance, which likely indicates the decomposition of the discussed complex.

Guanine, being a poorly soluble compound in water, shows relatively low absorbance compared to Adenine or the studied alkaloids. Three significant absorbance maxima were observed at approximately 220 nm, 250 nm, and 270 nm. However, when describing the spectra of the nedaplatin–guanine complexes, only the first wavelength was considered, which corresponds to approximately 220 nm. During the analysis of the UV-Vis spectroscopic spectrum of the nedaplatin–guanine complex, a second absorbance maximum was observed at about 270 nm, similar to what was noted for previously described complexes of the chemotherapeutic agent with Alkaloids and Adenine. For both absorbance maxima of the nedaplatin–guanine complexes, 220 nm and 270 nm, similar to the previously studied nedaplatin complexes with alkaloids and Adenine, an initial increase in absorbance was observed over time, followed by a decrease.

It should be emphasized that the experimentally obtained wavelengths for the absorbance maxima of nucleobases, for Adenine and Guanine, and alkaloid caffeine are the same as those found in many literature data sources [101,102,103,104,105].

Let us compare the theoretical and experimental excitation energies for the selected complexes (see Supplementary Materials). The concentration–absorbance experimental standard curves for the alkaloids Caffeine (Caf), Theobromine (Teb), and Theophylline (Tep) are shown in Figure S4 in the Supplementary Materials. The theoretical calculations were performed using the B3LYP/6-31G(d,p)/LANL2DZ and MN15/def2-TZVP methods. In the first case, the theoretical findings regarding the most intense singlet-singlet transitions for the nedaplatin-adenine complexes (N1-A, N2-A, and N3-A) are 293.2 nm, 309.6 nm, and 283.2 nm, respectively. When applying the method with greater accuracy, the values of the wavelengths for the absorbance maxima shifted slightly towards shorter wavelengths, yielding 272.8 nm, 289.7 nm, and 263.8 nm, respectively. The experimental data indicated that the most intense bands occurred at about 220 nm and 260 nm. For the complex with Guanine, the computed values at the B3LYP level are 295.2 nm for N1-G, 307.2 nm for N2-G, and 274.4 nm for N3-G, while the experimental spectra showed three absorbance maxima at approximately 220 nm, 250 nm, and 270 nm. The application of the MN15 method, similar to that for Adenine, resulted in slightly decreased values of 275.7 nm, 296 nm, and 268.7 nm, respectively. For the complexes with Caffeine, the most intense transitions were observed at 296 nm (N1-Caf), 305.6 nm (N2-Caf), and 284.8 nm (N3-Caf) using the B3LYP/6-31G(d,p)/LANL2DZ method, while the MN15/def2-TZVP method yielded values of 279.9 nm, 296 nm, and 272.9 nm. The experimental findings showed two absorption maxima at approximately 220 nm and 260 nm. The computed values for the Nedaplatin complexes (N1, N2, N3) with Theobromine are as follows: 296.8 nm, 254.8 nm, and 287.2 nm for the first level of calculations, and 276.4 nm, 296.7 nm, and 275 nm for the second theoretical level. The experimental data indicated absorption maxima at 223 nm and 272 nm, respectively. For the Nedaplatin complexes with Theophylline, we obtained values of 296.8 nm, 308 nm, and 284.8 nm (N1-Tep, N2-Tep, N3-Tep) for the first level of calculations, and 275 nm, 296 nm, and 272.2 nm (N1-Tep, N2-Tep, N3-Tep) for the MN15 method, which aligns well with the experimental data showing maximum absorbance bands at 269 nm and 227 nm. The computed UV-Vis spectra are presented in Figures S5–S10 in the Supplementary Material.

The comparison demonstrates a strong agreement between the computed spectra obtained using the MN15/def2-TZVP level of theory, employing the Polarizable Continuum Model (PCM) with water as a solvent, and the experimental spectra. A mean absolute deviation (MAD) analysis between the theoretical and experimental λ_max values was conducted (Table S1 in the Supplementary Materials). When all calculated and experimental wavelengths were considered, the MAD was 20.27 nm for the B3LYP method and 15.94 nm for MN15. In contrast, when only the closest matching theoretical and experimental values were compared, the MAD decreased to 4.92 nm for B3LYP and 2.66 nm for MN15. Overall, the MN15 method demonstrated better consistency with experimental results (Table S1 in the Supplementary Materials). A comparison of the calculated maximum absorbance wavelengths (λ) in nanometers (nm) for selected Nedaplatin complexes with nucleobases Adenine (A) and Guanine (G), as well as alkaloids such as Caffeine (Caf), Theobromine (Teb), and Theophylline (Tep), conducted using the B3LYP/6-31G(d,p)/LANL2DZ and MN15/def2-TZVP levels of theory with the PCM model and water as the solvent, can be found in Figure S11 (Supplementary Material). Additionally, the comparison of experimental maximum absorbance wavelength (λ) values (in nm) for selected substrates of complexation Adenine (A), Guanine (G), Caffeine (Caf), Theobromine (Teb), Theophylline (Tep), and Nedaplatin is shown in Figure S12. It should be noted, however, that an important challenge we encountered during the quantification of complex formation was the significant overlap of absorption spectra of alkaloids and their complexes with Nedaplatin in the UV-Vis range. This overlap made it difficult to accurately determine the equilibrium constants and reaction kinetics, as it was hard to clearly distinguish signals originating from free ligands and those associated with complexes.

This inherent limitation of UV-Vis spectroscopy, especially in the low UV range where both free ligands and complexes absorb, affects the precision of quantifying complex formation. As such, our conclusions regarding binding affinity are based primarily on qualitative spectral shifts and absorbance changes over time, rather than precise stoichiometric quantification. Future studies using complementary techniques such as NMR or mass spectrometry are warranted to overcome this limitation.

## 4. Conclusions

The theoretical investigation into the interactions of Nedaplatin with nucleobases and purine alkaloids, conducted using two levels of computational theory (B3LYP/6-31G(d,p)/LANL2DZ and MN15/def2-TZVP), has provided significant insights into the binding mechanisms and potential therapeutic implications of this chemical compound. The analysis reveals that Nedaplatin readily forms complexes with nucleobases, such as Adenine and Guanine, as well as with purine alkaloids, including Caffeine, Theobromine, and Theophylline. The primary binding interactions are attributed to the nitrogen atoms interacting with the platinum atom in Nedaplatin.

Calculations of Gibbs Free Energy change (ΔG_rs_) indicate that Nedaplatin shows a higher affinity for nucleobases compared to purine alkaloids, supporting the hypothesis of preferential binding. Among the nucleobases tested, Guanine emerged as the most favorable binding partner for Nedaplatin, suggesting its crucial role in enhancing the drug’s therapeutic effectiveness. The stability of complexes formed with nucleobases implies that these interactions may improve drug efficacy in cancer treatment.

Experimental studies utilizing UV-Vis spectroscopy corroborated the theoretical findings, confirming the formation of Nedaplatin-alkaloid complexes under physiological conditions (37 °C, pH 7.4). The concentration of these complexes increased over time, peaking after 36 h; however, they showed instability over extended periods, leading to eventual disintegration. The qualitative agreement between the calculated and experimental absorption spectra further validates the computational results.

The study also highlighted the variability in ΔG_rs_ values across different forms of Nedaplatin and computational methods, revealing complex interactions that may influence drug affinity. Notably, according to B3LYP calculations, the N2 hydrolysis product of Nedaplatin was identified as the most active form, while MN15 calculations indicated that both N2 and N3 forms exhibited the highest affinity for nucleobases and purine alkaloids, suggesting their equal favorability. The effects of increasing computational levels on the affinity of different Nedaplatin forms were variable: for instance, the affinity for N1 decreased, while it increased for N3 and remained relatively unchanged for N2.

Interestingly, despite the variations in ΔG_rs_ values for the alkaloids depending on the Nedaplatin form and the computational method, the binding affinities for Caffeine, Theophylline, and Theobromine remained nearly indistinguishable, as evidenced by their very low ΔΔG_rs_ values. This suggests that Nedaplatin maintains a consistent binding affinity across all tested alkaloids, a finding that is supported by experimental studies.

In conclusion, this research provides valuable insights into the bonding possibilities of Nedaplatin with aromatic compounds other than DNA nucleobases and emphasizes the potential implications of these interactions for enhancing future therapeutic strategies in chemotherapy. The findings of this research enhance our understanding of Nedaplatin interactions, setting the stage for further exploration of dietary purine alkaloids in cancer treatment. The use of various computational methods facilitated a detailed analysis, allowing for a robust comparison between theoretical predictions and experimental data, thereby strengthening the reliability of the results.

While the results of this study suggest that Nedaplatin is capable of forming stable complexes with purine alkaloids such as Caffeine, Theobromine, and Theophylline, these findings should be interpreted as preliminary. Although dietary exposure to these alkaloids is widespread, no current clinical or in vivo data confirm that such interactions significantly impact Nedaplatin’s therapeutic activity. Therefore, the observed molecular interactions should be considered as a basis for hypothesis generation rather than evidence of clinically relevant drug–diet interactions. Further studies, including pharmacokinetic and in vivo models, are necessary to explore this possibility.

## Figures and Tables

**Table 1:** Values of the change in Gibbs Free Energy of reaction (ΔG_rs_) for the complexations of N1, N2 and N3 with purine alkaloids and nucleobases at two levels of calculations, B3LYP/6-31G(d,p)/LANL2DZ and MN15/def2-TZV.

Reaction	ΔG_rs_ [kcal/mol]	
	B3LYP/6-31G(d,p)/LANL2DZ	MN15/def2-TZV
N1 + A → N1-A	−20.6	−15.3
N1 + G → N1-G	−22.9	−17.8
N1 + Caf → N1-Caf	−14.7	−10.6
N1 + Teb → N1-Teb	−15.7	−10.7
N1 + Tep → N1-Tep	−15.3	−9.7
N2 + A → N2-A	−27.2	−27.1
N2 + G → N2-G	−33.3	−31.2
N2 + Caf → N2-Caf	−19.1	−21.2
N2 + Teb → N2-Teb	−18.7	−21.4
N2 + Tep → N2-Tep	−19.8	−21.0
N3 + A → N3-A	−19.3	−28.5
N3 + G → N3-G	−23.3	−28.0
N3 + Caf → N3-Caf	−14.4	−23.1
N3 + Teb → N3-Teb	−14.4	−22.6
N3 + Tep → N3-Tep	−14.6	−22.8

## Data Availability

Requests for further information and resources should be directed to and will be fulfilled by the lead contact, Beata Szefler (beatas@cm.umk.pl). This study did not generate new unique reagents.

## Supplementary Materials

The following supporting information can be downloaded at: https://www.mdpi.com/article/10.3390/biomedicines13071551/s1. Figure S1. Structures of Nedaplatin complexes with nucleobases (Adenine (A), Guanine (G)) and alkaloids (Caffeine (Caf), Theobromine (Teb), Theophylline (Tep)) computed using the B3LYP/6-31G(d,p)/LANL2DZ and MN15/def2-TZVP levels of theory, employing the PCM model with water as the solvent. Figure S2. Changes in the maximum absorbance over time for selected Nedaplatin complexes with the nucleobases Adenine (A) and Guanine (G) in experimental UV-Vis spectra. Figure S3. Changes in the time of maximum absorbance for selected Nedaplatin complexes with alkaloids, including Caffeine (Caf), Theobromine (Teb), and Theophylline (Tep), as observed in experimental UV-Vis spectra. Figure S4. Standard curves for alkaloids: Caffeine (Caf), Theobromine (Teb), and Theophylline (Tep). Experimental data from UV-Vis spectroscopy study. Figure S5. Computed UV-Vis spectra for selected nucleobases—Adenine (A) and Guanine (G)—as well as selected alkaloids: Caffeine (Caf), Theobromine (Teb), and Theophylline (Tep). These calculations were conducted after optimization at the B3LYP/6-31G(d,p) LANL2DZ level of theory, employing the PCM model with water as the solvent. All spectroscopic calculations utilized the PBE0 functional. Figure S6. Computed UV-Vis spectra for selected Nedaplatin (N1, N2, N3 hydrolysis products) complexes with the nucleobases Adenine (A) and Guanine (G), optimized at the B3LYP/6-31G(d,p)/LANL2DZ level of theory using the PCM model with water as the solvent. All spectroscopic calculations were carried out using the PBE0 functional. Figure S7. Computed UV-Vis spectra for selected Nedaplatin (N1, N2, N3 hydrolysis products) complexes with alkaloids, including Caffeine (Caf), Theobromine (Teb), and Theophylline (Tep), after optimization at the B3LYP/6-31G(d,p)/LANL2DZ level of theory using the PCM model with water as the solvent. All spectroscopic calculations were carried out using the PBE0 functional using the PBE0 functional. Figure S8. Computed UV-Vis spectra for selected nucleobases Adenine (A), Guanine (G) and alkaloids Caffeine (Caf), Theobromine (Teb), Theophylline (Tep) after optimization at MN15/def2-TZVP level of theory with PCM model and water as a solvent. All spectroscopic calculations were performed using PBE0 functional. Figure S9. Computed UV-Vis spectra for selected Nedaplatin (N1, N2, N3 products of hydrolysis) complexes with nucleobases Adenine (A), Guanine (G) after optimization at MN15/def2-TZVP level of theory with PCM model and water as a solvent. All spectroscopic calculations were performed using PBE0 functional. Figure S10. Computed UV-Vis spectra for selected Nedaplatin (N1, N2, N3 products of hydrolysis) complexes with alkaloids, such as Caffeine (Caf), Theobromine (Teb), Theophylline (Tep) after optimization at MN15/def2-TZVP level of theory with PCM model and water as a solvent. All spectroscopic calculations were performed using PBE0 functional. Figure S11. Comparison of calculated maximum absorbance wavelengths (λ) in nanometers (nm) for selected Nedaplatin complexes with nucleobases Adenine (A) and Guanine (G), as well as alkaloids such as Caffeine (Caf), Theobromine (Teb), and Theophylline (Tep). The calculations were performed using B3LYP/6-31G(d,p)/LANL2DZ and MN15/def2-TZVP levels of theory with a PCM model and water as the solvent. Figure S12. Comparison of experimental maximum Absorbance wavelength (λ) values (in nm) for selected substrates of complexations such as Adenine (A), Guanine (G) Caffeine (Caf), Theobromine (Teb), Theophylline (Tep) and Nedaplatin. Table S1. Comparison of Experimental and Theoretical λmax Values using Mean Absolute Deviation (MAD).
