# Supplementary material for: Interactions of Nedaplatin with Nucleobases and Purine Alkaloids: Their Role in Cancer Therapy
Source: Biomedicines. 2025 Jun 25;13(7):1551. doi: 10.3390/biomedicines13071551 (PMC12292643; doi:10.3390/biomedicines13071551)
Supplement: Supplementary file 1 [file biomedicines-13-01551-s001.zip › biomedicines-3687471-supplementary.pdf]

Interactions of Nedaplatin with Nucleobases and Purine Alkaloids: Their Role in Cancer Therapy

Kamil Szupryczyński [1] and Beata Szefler [2,*]

[1] *Doctoral School of Medical and Health Sciences, Faculty of Pharmacy, Collegium Medicum, Nicolaus Copernicus University, Jagiellońska 13, 85-067 Bydgoszcz, Poland; 503592@doktorant.umk.pl*
[2] *Department of Physical Chemistry, Faculty of Pharmacy, Collegium Medicum, Nicolaus Copernicus University, Kurpińskiego 5, 85-096 Bydgoszcz, Poland*
* *Correspondence: beatas@cm.umk.pl*

Table of contents:

Figure S1. *Structures of nedaplatin complexes with nucleobases (Adenine (A), Guanine (G)) and alkaloids (Caffeine (Caf), Theobromine (Teb), Theophylline (Tep)) computed using the B3LYP/6-31G(d,p)/LANL2DZ and MN15/def2-TZVP levels of theory, employing the PCM model with water as the solvent.*

Figure S2. *Changes in the maximum absorbance over time for selected Nedaplatin complexes with the nucleobases Adenine (A) and Guanine (G) in experimental UV-Vis spectra.*

Figure S3. *Changes in the time of maximum absorbance for selected Nedaplatin complexes with alkaloids, including Caffeine (Caf), Theobromine (Teb), and Theophylline (Tep), as observed in experimental UV-Vis spectra.*

Figure S4. *Standard curves for alkaloids: Caffeine (Caf), Theobromine (Teb), and Theophylline (Tep). Experimental data from UV-Vis spectroscopy study.*

Figure S5. *Computed UV-Vis spectra for selected nucleobases—Adenine (A) and Guanine (G)—as well as selected alkaloids: Caffeine (Caf), Theobromine (Teb), and Theophylline (Tep). These calculations were conducted after optimization at the B3LYP/6-31G(d,p) LANL2DZ level of theory, employing the PCM model with water as the solvent. All spectroscopic calculations utilized the PBE0 functional.*

Figure S6. *Computed UV-Vis spectra for selected Nedaplatin (N1, N2, N3 hydrolysis products) complexes with the nucleobases Adenine (A) and Guanine (G), optimized at the B3LYP/6-31G(d,p)/LANL2DZ level of theory using the PCM model with water as the solvent. All spectroscopic calculations were carried out using the PBE0 functional.*

Figure S7. *Computed UV-Vis spectra for selected Nedaplatin (N1, N2, N3 hydrolysis products) complexes with alkaloids, including Caffeine (Caf), Theobromine (Teb), and Theophylline (Tep), after optimization at the B3LYP/6-31G(d,p)/LANL2DZ level of theory using the PCM model with water as the solvent. All spectroscopic calculations were carried out using the PBE0 functional.*

Figure S8. *Computed UV-Vis spectra for selected nucleobases Adenine (A), Guanine (G) and alkaloids Caffeine (Caf), Theobromine(Teb), Theophylline (Tep) after optimization at MN15/def2-TZVP level of theory with PCM model and water as a solvent. All spectroscopic calculations were performed using PBE0 functional.*

Figure S9. *Computed UV-Vis spectra for selected Nedaplatin (N1, N2, N3 products of hydrolysis) complexes with nucleobases Adenine (A), Guanine (G) after optimization at MN15/def2-TZVP level of theory with PCM model and water as a solvent. All spectroscopic calculations were performed using PBE0 functional.*

Figure S10. *Computed UV-Vis spectra for selected Nedaplatin (N1, N2, N3 products of hydrolysis) complexes with alkaloids, such as Caffeine (Caf), Theobromine(Teb), Theophylline (Tep) after optimization at MN15/def2-TZVP level of theory with PCM model and water as a solvent. All spectroscopic calculations were performed using PBE0 functional.*

Figure S11. *Comparison of calculated maximum absorbance wavelengths (λ) in nanometers (nm) for selected Nedaplatin complexes with nucleobases Adenine (A) and Guanine (G), as well as alkaloids such as Caffeine (Caf), Theobromine (Teb), and Theophylline (Tep). The calculations were performed using B3LYP/6-31G(d,p)/LANL2DZ and MN15/def2-TZVP levels of theory with a PCM model and water as the solvent.*

Figure S12. *Comparison of experimental maximum Absorbance wavelength (λ) values (in nm) for selected substrates of complexations such as Adenine (A), Guanine (G) Caffeine (Caf), Theobromine(Teb), Theophylline (Tep) and Nedaplatin.*

Table S1. *Comparison of Experimental and Theoretical λmax Values using Mean Absolute Deviation (MAD).*

<table>
<tr><td colspan="2" rowspan="2"></td><td colspan="3">Substrate of complexation</td></tr>
<tr><td>N1
product of hydrolysis</td><td>N2
product of hydrolysis</td><td>N3
product of hydrolysis</td></tr>
<tr><td rowspan="3">Subs
trate
of
com
plex
ation</td><td rowspan="2">Ade
nine
(A)</td><td>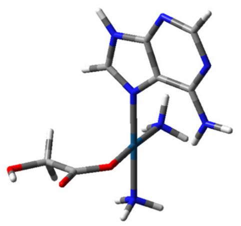</td><td>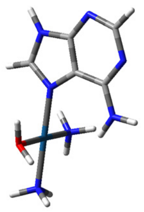</td><td>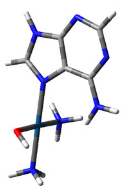</td></tr>
<tr><td>N1-A</td><td>N2-A</td><td>N3-A</td></tr>
<tr><td>Gua
nine
(G)</td><td>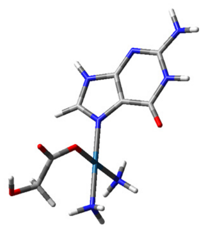</td><td>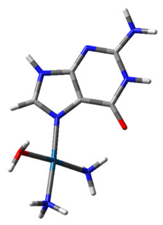</td><td>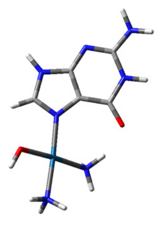</td></tr>
</table>

		N1-G	N2-G	N3-G
	Caffeine (Caf)	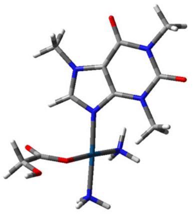	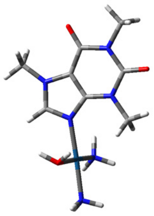	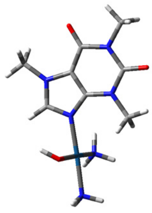
		N1-Caf	N2-Caf	N3-Caf
	Theophylline (Tep)	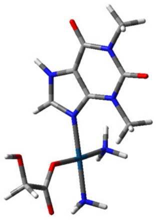	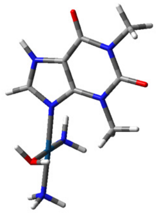	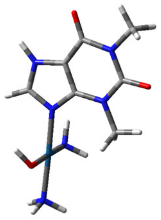
		N1-Tep	N2-Tep	N3-Tep
	Theobromine (Teb)	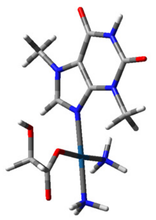	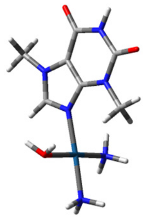	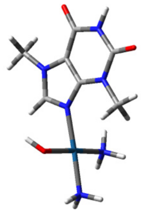
		N1-Teb	N2-Teb	N3-Teb

Figure S1. *Structures of Nedaplatin complexes with nucleobases (Adenine (A), Guanine (G)) and alkaloids (Caffeine (Caf), Theobromine (Teb), Theophylline (Tep)) computed using the B3LYP/6-31G(d,p)/LANL2DZ and MN15/def2-TZVP levels of theory, employing the PCM model with water as the solvent. Experimental data from UV-Vis spectroscopy study. The nitrogen atom (N) is marked in blue, the oxygen atom (O) in red, and the carbon atom (C) in gray, respectively.*

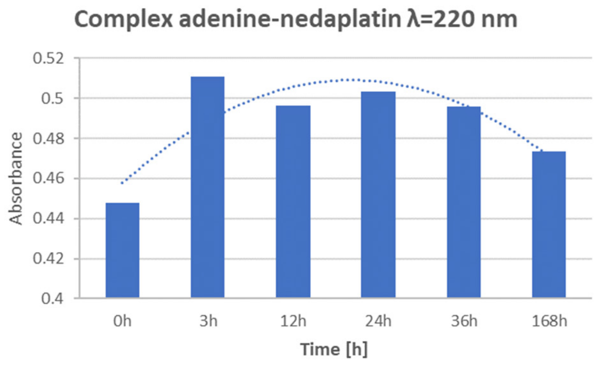

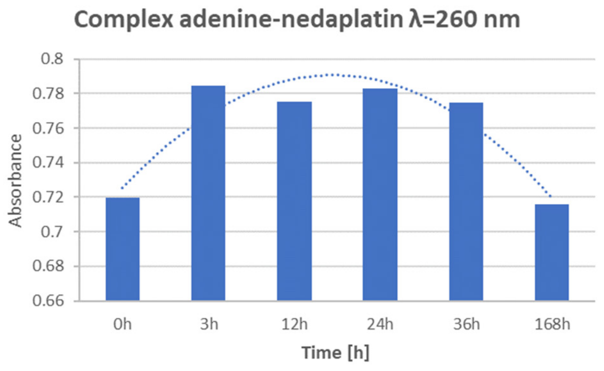

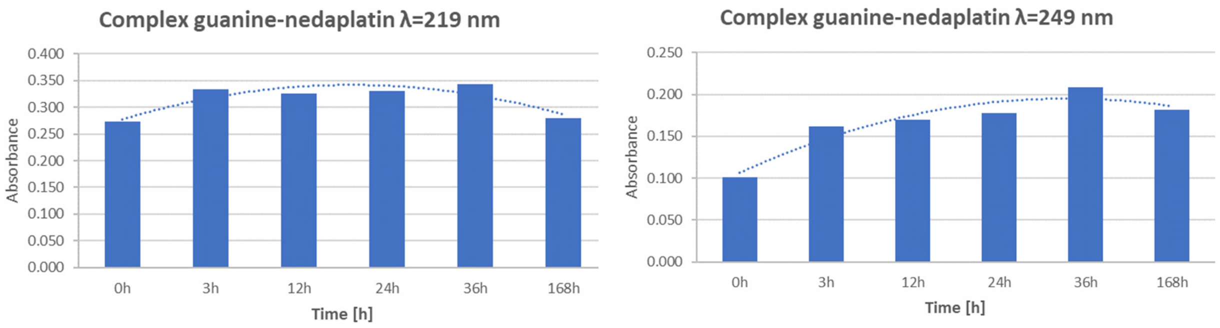

Figure S2. *Changes in the maximum absorbance over time for selected Nedaplatin complexes with the nucleobases Adenine (A) and Guanine (G) in experimental UV-Vis spectra.*

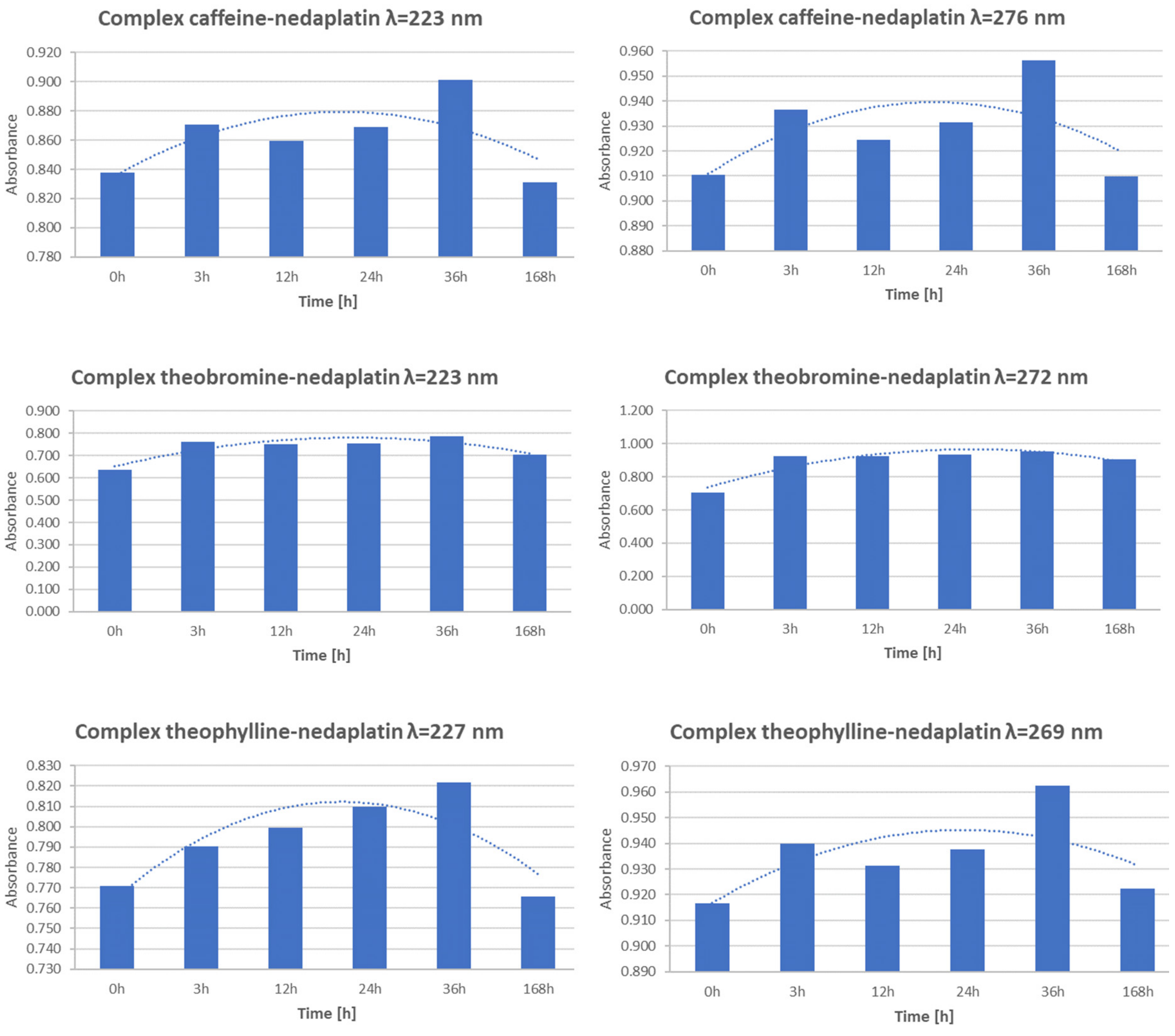

Figure S3. *Changes in the time of maximum absorbance for selected Nedaplatin complexes with alkaloids, including Caffeine (Caf), Theobromine (Teb), and Theophylline (Tep), as observed in experimental UV-Vis spectra.*

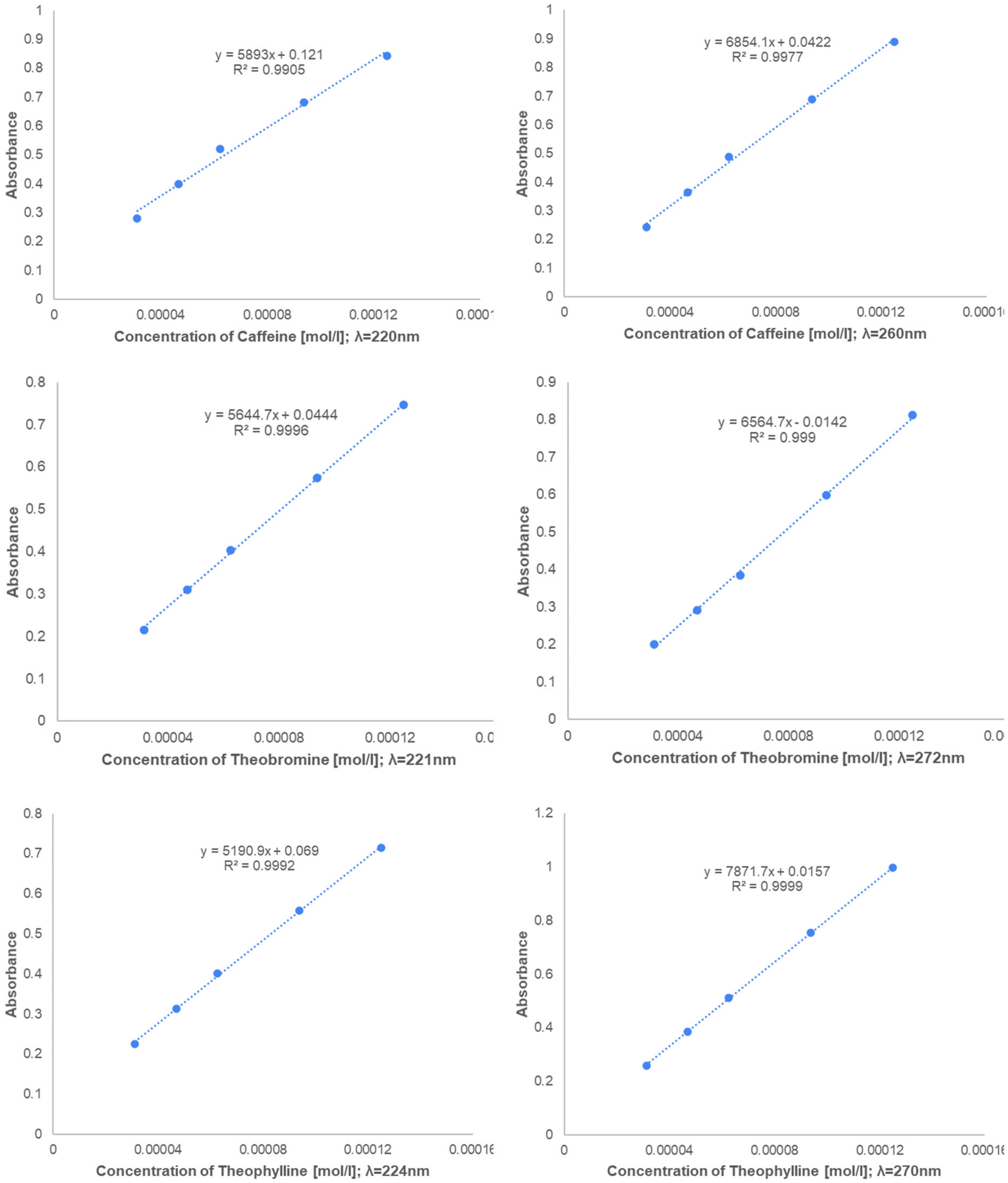

Figure S4. *Standard curves for alkaloids: Caffeine (Caf), Theobromine (Teb), and Theophylline (Tep). Experimental data from UV-Vis spectroscopy study.*

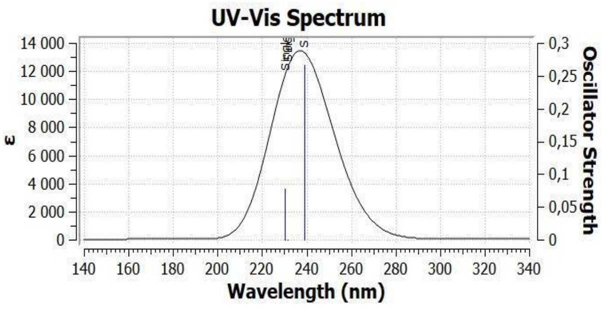

Adenine (A)

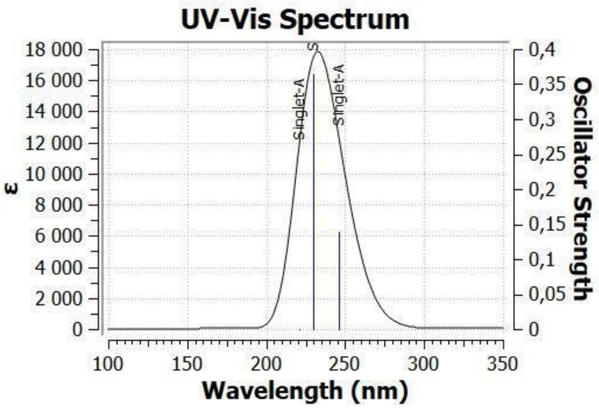

Guanine (G)

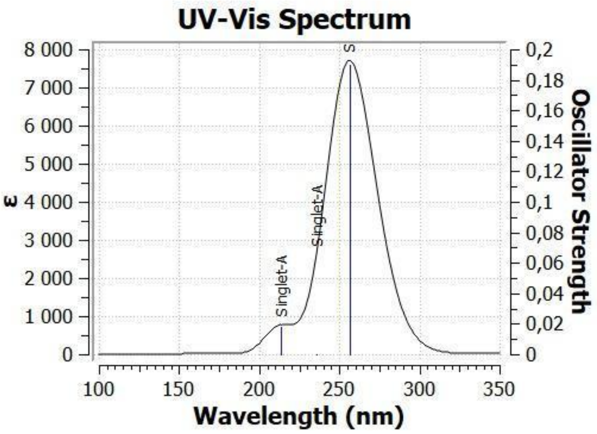

Caffeine (Caf)

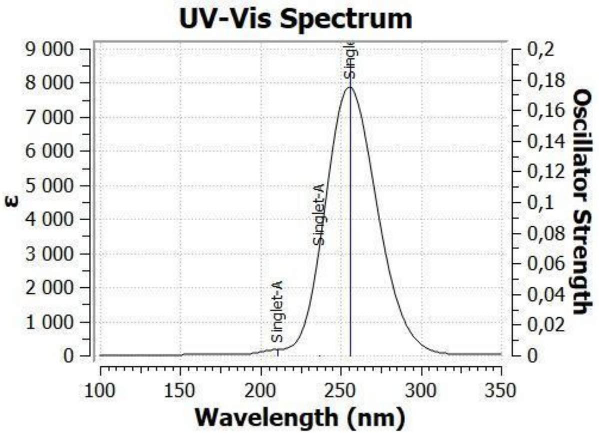

Teobromine (Teb)

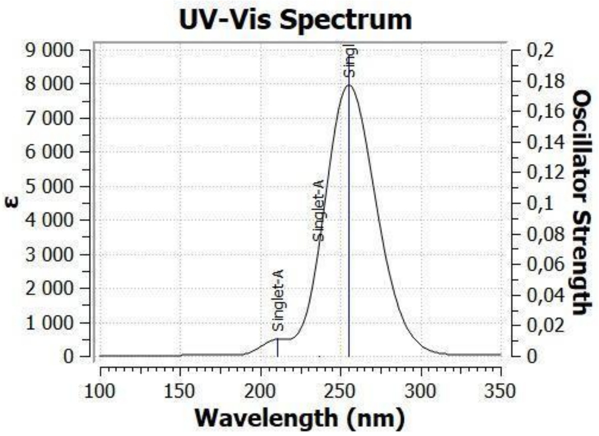

Theophylline (Tep)

***Figure S5** Computed UV-Vis spectra for selected nucleobases—Adenine (A) and Guanine (G)—as well as selected alkaloids: Caffeine (Caf), Theobromine (Teb), and Theophylline (Tep). These calculations were conducted after optimization at the B3LYP/6-31G(d,p) LANL2DZ level of theory, employing the PCM model with water as the solvent. All spectroscopic calculations utilized the PBE0 functional.*

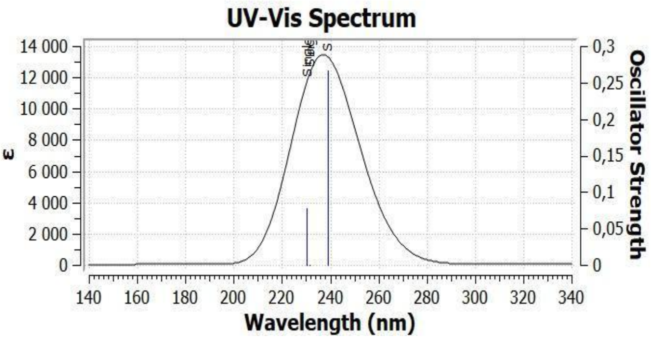

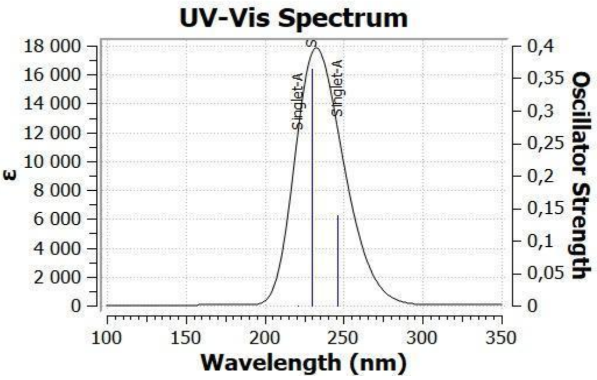

N1-A

N1-G

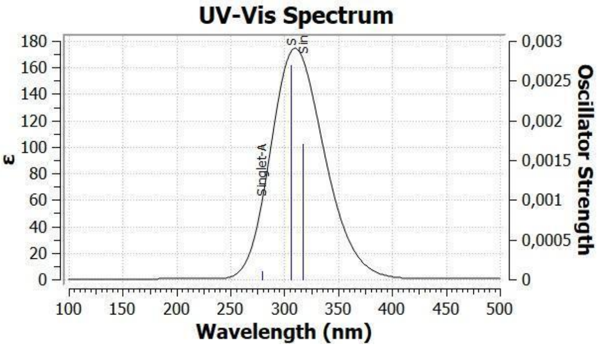

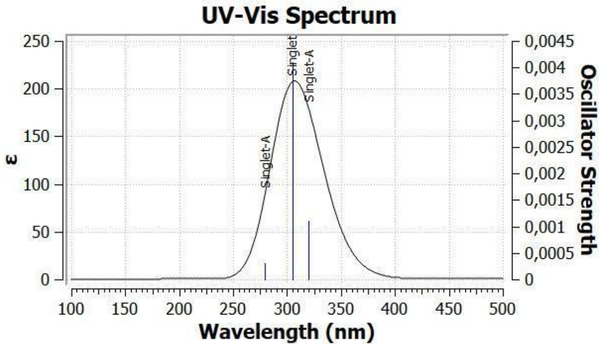

N2-A

N2-G

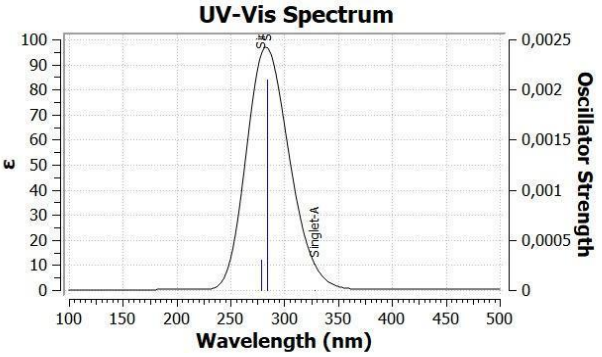

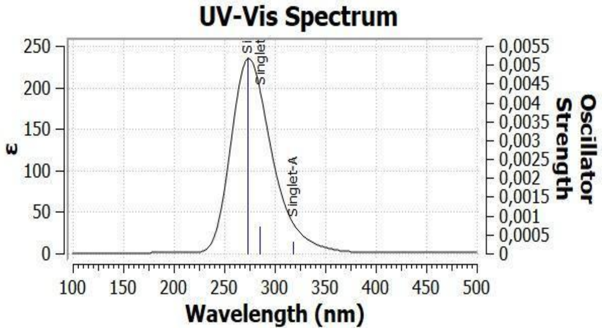

N3-A

N3-G

Figure S6. *Computed UV-Vis spectra for selected Nedaplatin (N1, N2, N3 hydrolysis products) complexes with the nucleobases Adenine (A) and Guanine (G), optimized at the B3LYP/6-31G(d,p)/LANL2DZ level of theory using the PCM model with water as the solvent. All spectroscopic calculations were carried out using the PBE0 functional.*

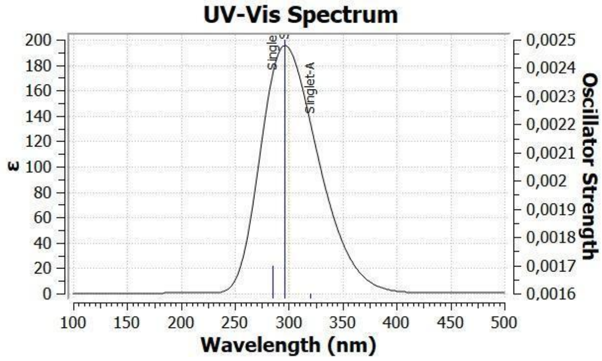

N1-Caf

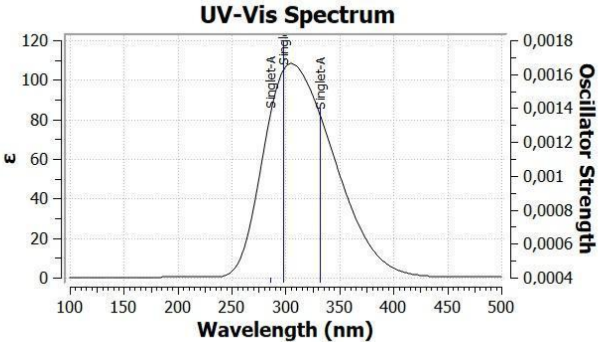

N2-Caf

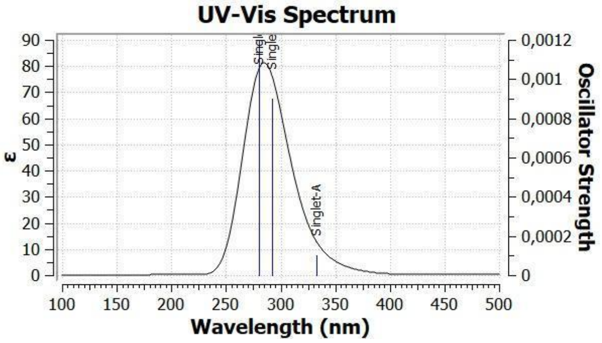

N3-Caf

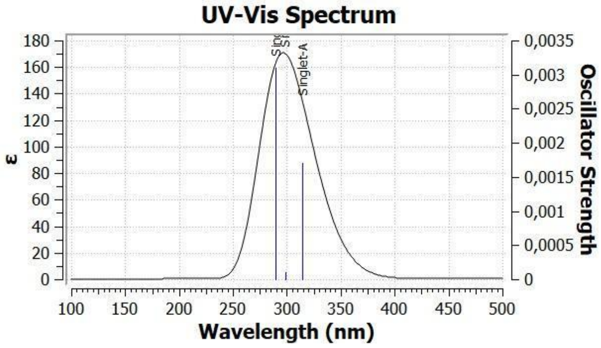

N1-Teb

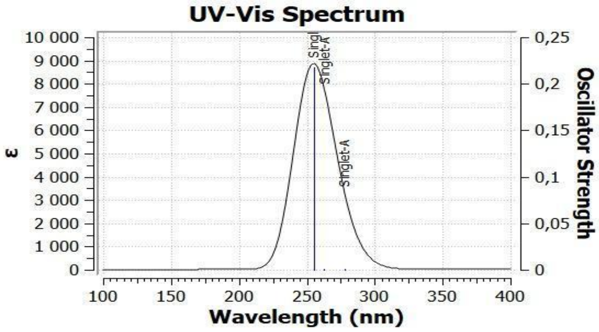

N2- Teb

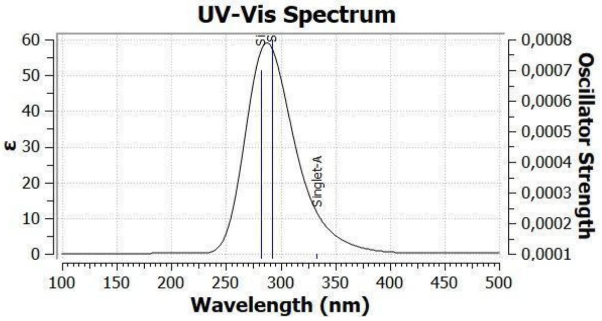

N3- Teb

Figure S7. *Computed UV-Vis spectra for selected Nedaplatin (N1, N2, N3 hydrolysis products) complexes with alkaloids, including Caffeine (Caf), Theobromine (Teb), and Theophylline (Tep), after optimization at the B3LYP/6-31G(d,p)/LANL2DZ level of theory using the PCM model with water as the solvent. All spectroscopic calculations were carried out using the PBE0 functional.*

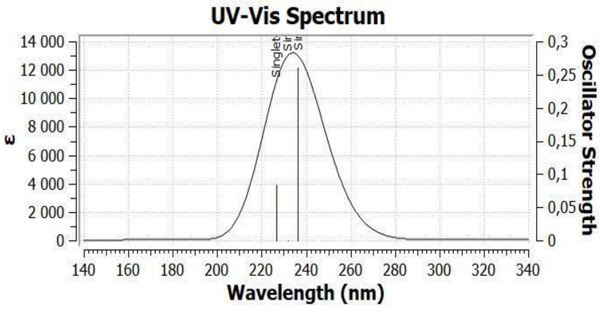

Adenine (A)

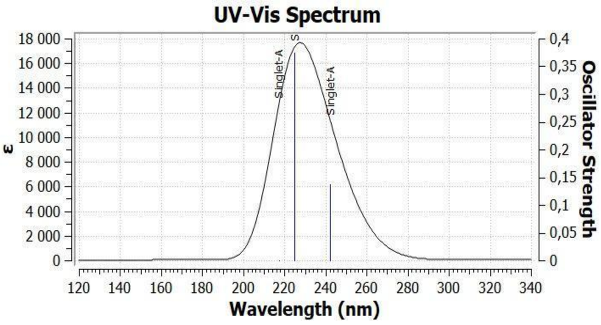

Guanine (G)

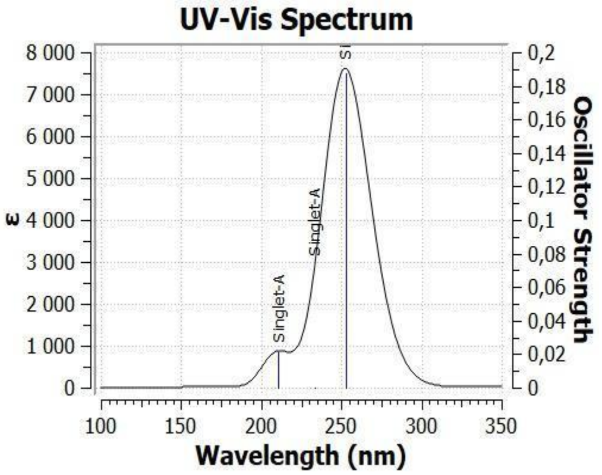

Caffeine (Caf)

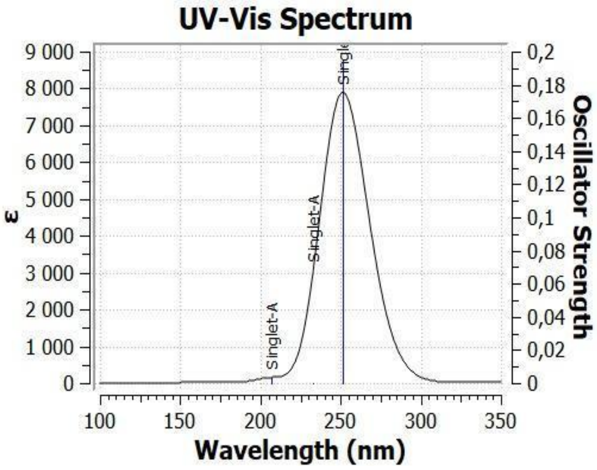

Teobromine (Teb)

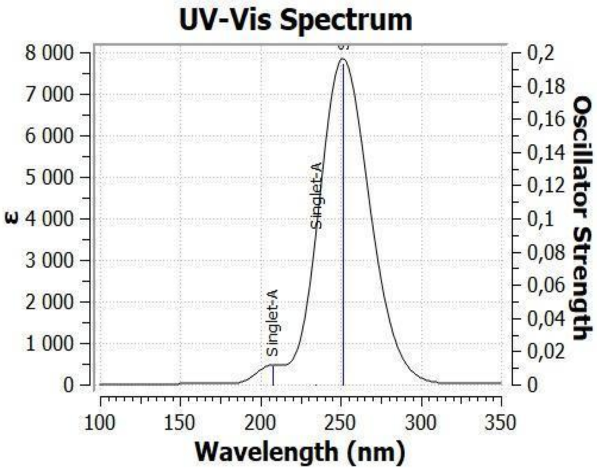

Theophylline (Tep)

Figure S8. *Computed UV-Vis spectra for selected nucleobases Adenine (A), Guanine (G) and alkaloids Caffeine (Caf), Theobromine(Teb), Theophylline (Tep) after optimization at MN15/def2-TZVP level of theory with PCM model and water as a solvent. All spectroscopic calculations were performed using PBE0 functional.*

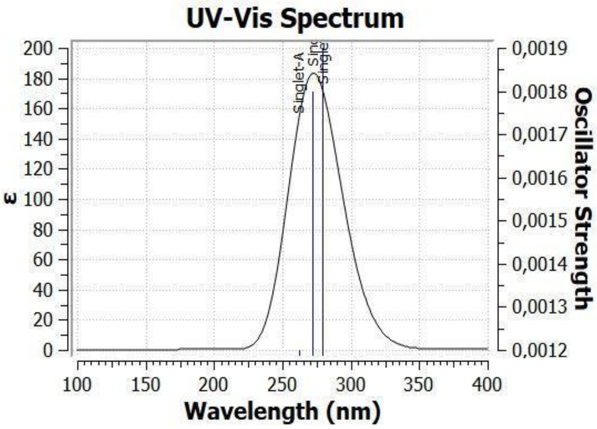

N1-A

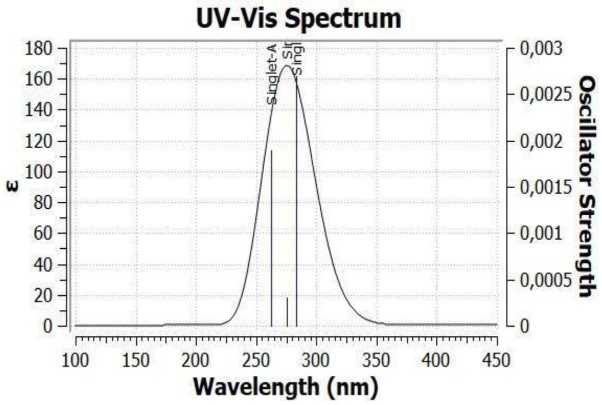

N1-G

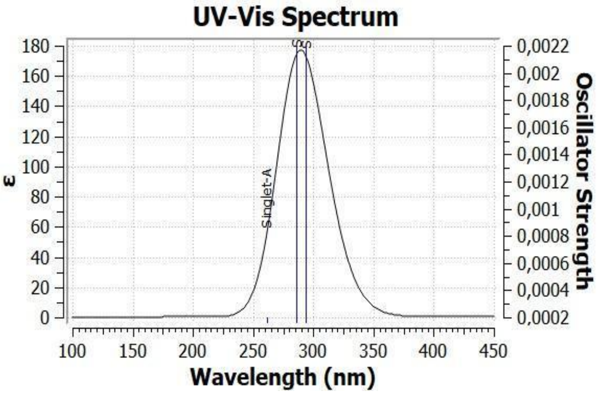

N2-A

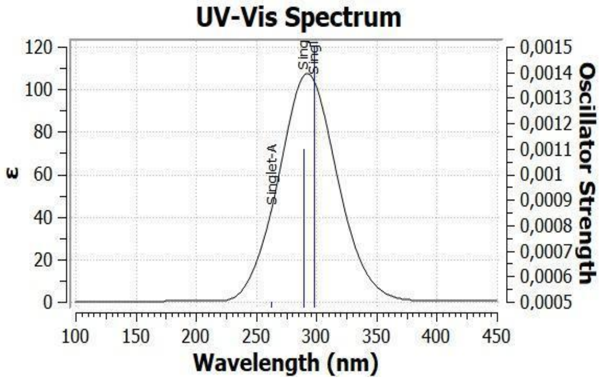

N2-G

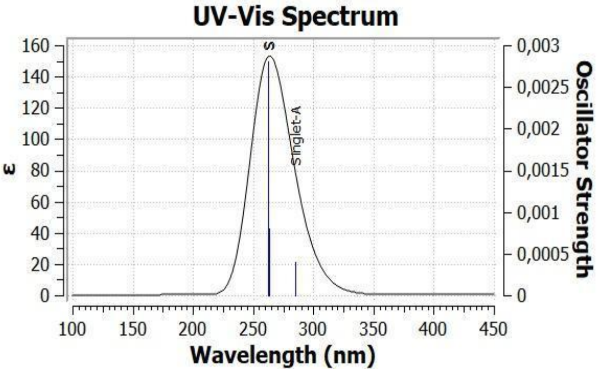

N3-A

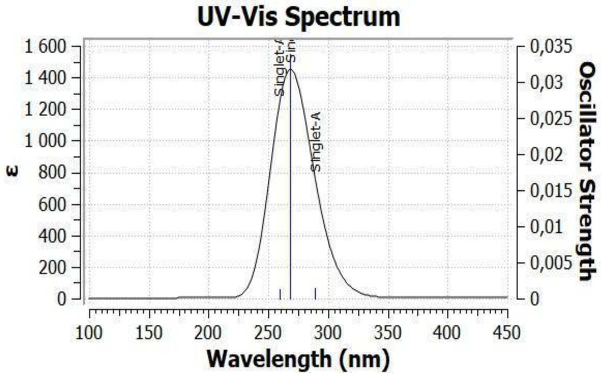

N3-G

Figure S9. *Computed UV-Vis spectra for selected Nedaplatin (N1, N2, N3 products of hydrolysis) complexes with nucleobases Adenine (A), Guanine (G) after optimization at MN15/def2-TZVP level of theory with PCM model and water as a solvent. All spectroscopic calculations were performed using PBE0 functional.*

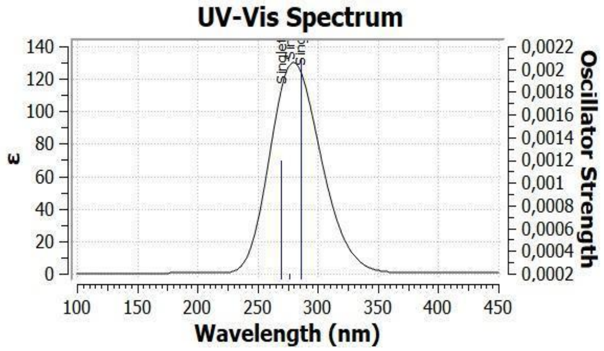

N1-Caf

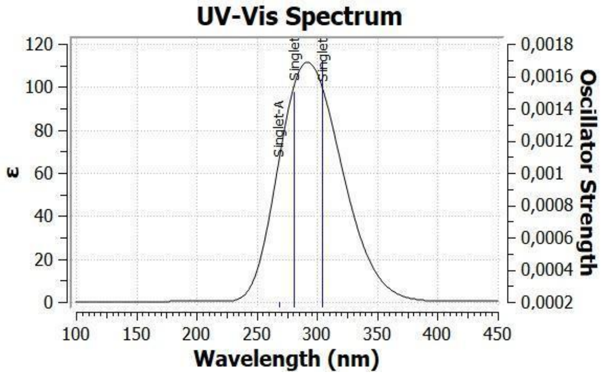

N2-Caf

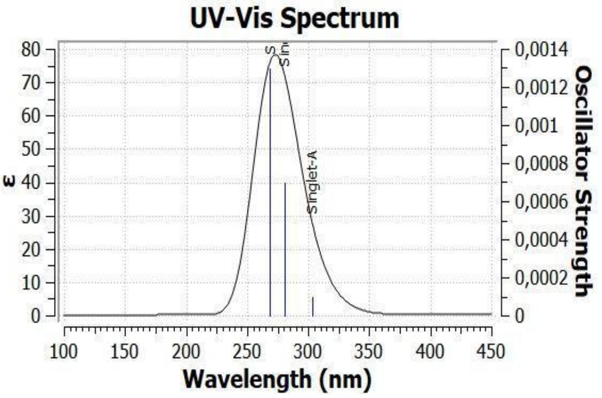

N3-Caf

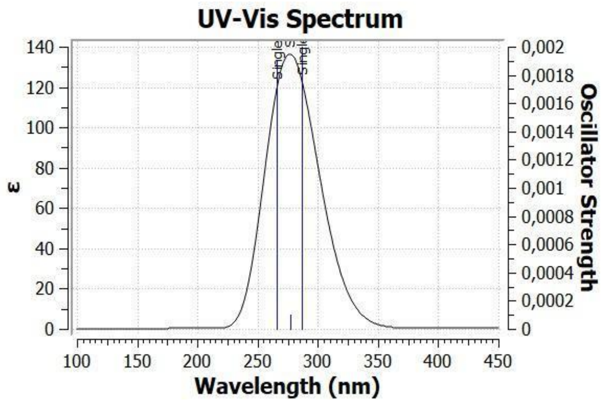

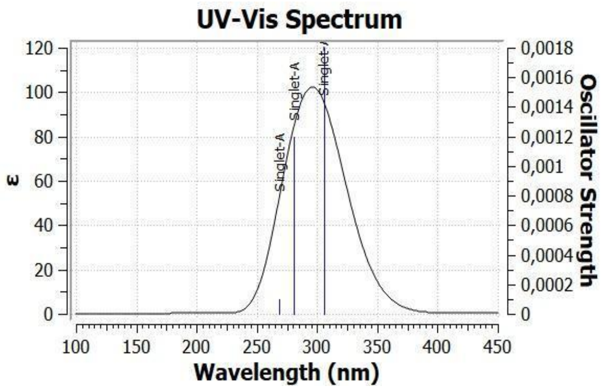

N1-Teb N2- Teb

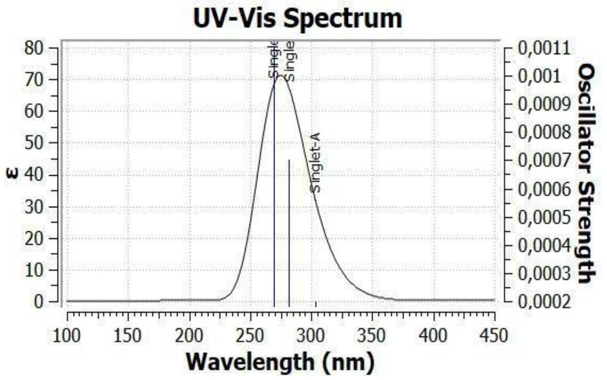

N3- Teb

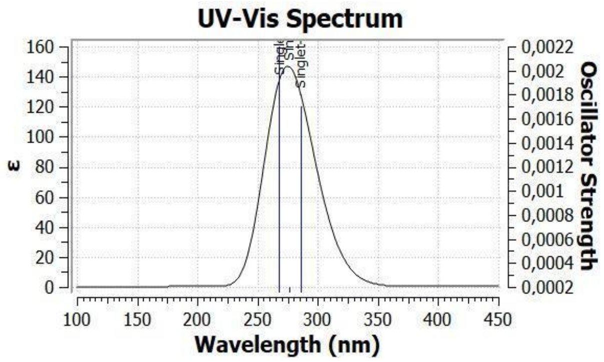

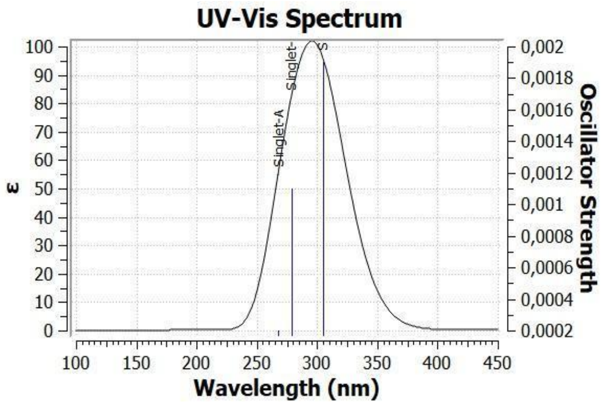

N1-Tep N2- Tep

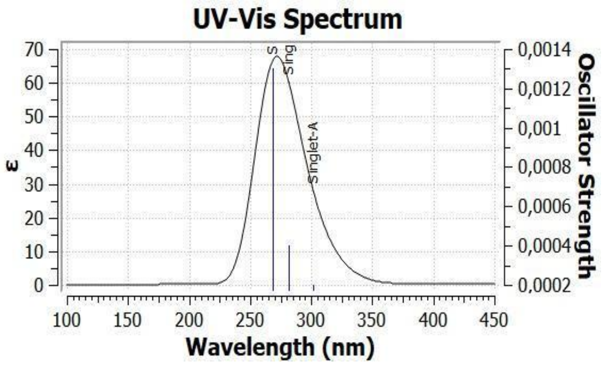

N3- Tep

Figure S10. *Computed UV-Vis spectra for selected Nedaplatin (N1, N2, N3 products of hydrolysis) complexes with alkaloids, such as Caffeine (Caf), Theobromine(Teb), Theophylline (Tep) after optimization at MN15/def2-*

TZVP level of theory with PCM model and water as a solvent. All spectroscopic calculations were performed using PBE0 functional.

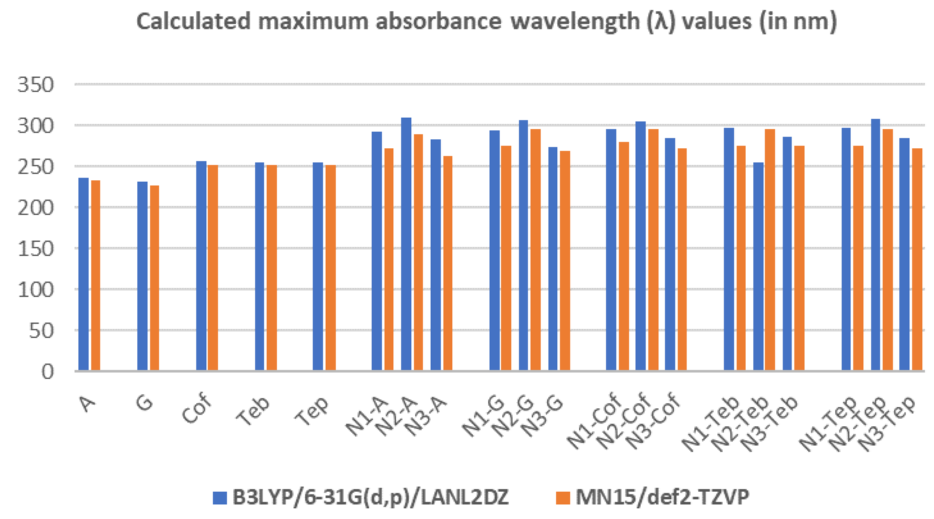

Figure S11. *Comparison of calculated maximum absorbance wavelengths (λ) in nanometers (nm) for selected Nedaplatin complexes with nucleobases Adenine (A) and Guanine (G), as well as alkaloids such as Caffeine (Caf), Theobromine (Teb), and Theophylline (Tep). The calculations were performed using B3LYP/6-31G(d,p)/LANL2DZ and MN15/def2-TZVP levels of theory with a PCM model and water as the solvent.*

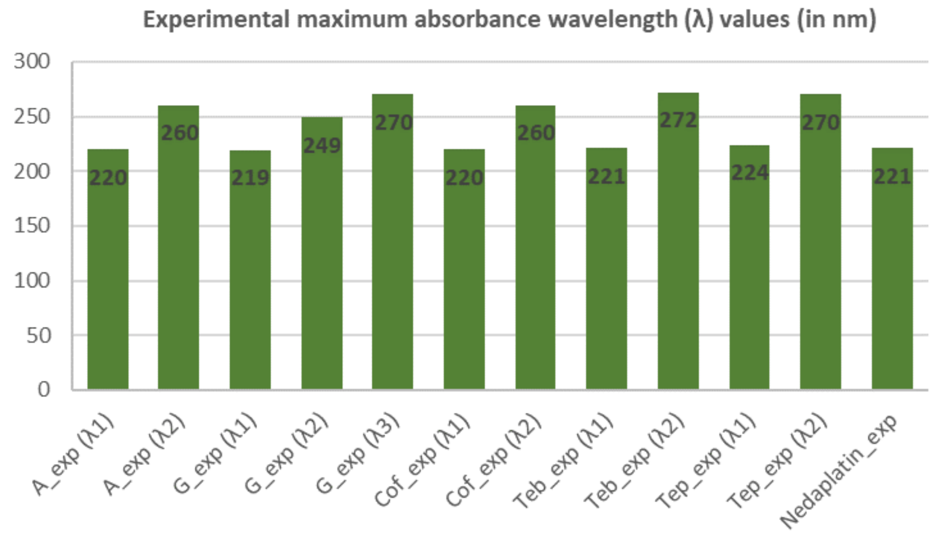

Figure S12. *Comparison of experimental maximum Absorbance wavelength (λ) values (in nm) for selected substrates of complexations such as Adenine (A), Guanine (G) Caffeine (Caf), Theobromine(Teb), Theophylline (Tep) and Nedaplatin.*

Table S1*. Comparison of Experimental and Theoretical λmax Values using Mean Absolute Deviation (MAD)[1–5].*

Compound	Experimental	B3LYP/6-31G(d,p)/LANL2DZ		MN15/def2-TZVP	
	**λmax [nm]**	**λmax [nm]**	**MAD**	**λmax [nm]**	**MAD**
**Adenine**	220.0	237.2	8.6	234	7.0
	260.0		11.4		13.0
**Guanine**	219.0	232.5	6.8	227.8	4.4
	249.0		8.3		10.6
	270.0		18.8		21.1
**Caffeine**	220.0	256.5	18.3	252.5	16.3
	260.0		1.8		3.8
**Theobromine**	221.0	255.5	17.3	251.5	17.2
	272.0		8.3		10.3
**Theophylline**	224.0	255.5	15.8	251.5	13.8
	270.0		7.3		16.9
**N1-A**	220.0	293.2	36.6	272.8	26.4
	260.0		16.6		6.4
**N1-G**	219.0	295.2	38.1	275.7	28.4
	249.0		23.1		13.4
	271.0		12.1		2.3
**N1-Caf**	223.0	296.0	36.5	279.9	28.5
	276.0		10.0		1.9
**N1-Teb**	223.0	296.8	36.9	276.4	26.7
	272.0		12.4		2.2
**N1-Tep**	227.0	296.8	34.9	275.0	24.0
	269.0		13.9		3.0
**N2-A**	220.0	309.6	44.8	289.7	34.9
	260.0		24.8		14.9
**N2-G**	219.0	307.2	44.1	296.0	38.5
	249 .0		29.1		23.5
	271.0		18.1		12.5
**N2-Caf**	223.0	305.6	41.3	296.0	36.5
	276.0		14.8		10.0
**N2-Teb**	223.0	254.8	15.9	296.7	36.9
	272.0		8.6		12.4
**N2-Tep**	227.0	308.0	40.5	296.0	34.5
	269.0		19.5		13.5
**N3-A**	220.0	283.2	31.6	263.8	21.9
	260.0		11.6		1.9
**N3-G**	219.0	274.4	27.7	268.7	24.9
	249 .0		12.7		9.8
	271.0		1.7		1.2
**N3-Caf**	223.0	284.8	30.9	272.9	25.0
	276.0		4.4		1.6
**N3-Teb**	223.0	287.2	32.1	275.0	26.0
	272.0		7.6		1.5
**N3-Tep**	227.0	284.8	28.9	272.2	22.6
	269.0		7.9		1.6

References

1. Fischer, J. Specific Detection of Nucleotides, Creatine Phosphate, and Their Derivatives from Tissue Samples in a Simple, Isocratic, Recycling, Low-Volume System. LC-GC Int. 1995, 8, 254–264.
2. Belay, A.; Ture, K.; Redi, M.; Asfaw, A. Measurement of Caffeine in Coffee Beans with UV/Vis Spectrometer. *Food Chem.* **2008**, *108*, 310–315, doi:10.1016/j.foodchem.2007.10.024.
3. Guerra, J.M.; Hernández, A.R.; Garrido, L.D.G.; Silva, M.T.R. New Insights about Equilibrium Constants, pKa, of Purine Nitrogenous Bases: The Case of Adenine and Guanine. A UV-Vis Spectrophotometric Study at I = 0.4 M. *J. Mex. Chem. Soc.* **2025**, *69*, 70–77, doi:10.29356/jmcs.v69i1.2292.
4. Barbatti, M.; Aquino, A.J.A.; Lischka, H. The UV Absorption of Nucleobases: Semi-Classical Ab Initio Spectra Simulations. *Phys. Chem. Chem. Phys.* **2010**, *12*, 4959–4967, doi:10.1039/B924956G.
5. Kaspar, F.; Giessmann, R.T.; Krausch, N.; Neubauer, P.; Wagner, A.; Gimpel, M. A UV/Vis Spectroscopy-Based Assay for Monitoring of Transformations Between Nucleosides and Nucleobases. *Methods Protoc.* **2019**, *2*, 60, doi:10.3390/mps2030060.
